# Quantum Dot-Enabled Biosensing for Prostate Cancer Diagnostics

**DOI:** 10.3390/nano15151162

**Published:** 2025-07-28

**Authors:** Hossein Omidian, Erma J. Gill, Luigi X. Cubeddu

**Affiliations:** Barry and Judy Silverman College of Pharmacy, Nova Southeastern University, Fort Lauderdale, FL 33328, USA; eg1262@mynsu.nova.edu

**Keywords:** quantum dots, prostate-specific antigen (PSA), biosensors, multiplex diagnostics, nanomaterials

## Abstract

Prostate cancer diagnostics are rapidly advancing through innovations in nanotechnology, biosensing strategies, and molecular recognition. This review analyzes studies focusing on quantum dot (QD)-based biosensors for detecting prostate cancer biomarkers with high sensitivity and specificity. It covers diverse sensing platforms and signal transduction mechanisms, emphasizing the influence of the QD composition, surface functionalization, and bio interface engineering on analytical performance. Key metrics such as detection limits, dynamic range, and compatibility with biological samples, including serum, urine, and tissue, are critically assessed. Recent advances in green-synthesized QDs and smartphone-integrated diagnostic platforms are highlighted, including lateral flow assays, paper-based devices, and pH-responsive hydrogels for real-time, low-cost, and decentralized cancer screening. These innovations enable multiplexed biomarker detection and tumor microenvironment monitoring in point-of-care settings. This review concludes by addressing the current limitations, scalability challenges, and future research directions for translating QD-enabled biosensors into clinically viable diagnostic tools.

## 1. Introduction

Prostate cancer (PCa) remains one of the most frequently diagnosed malignancies in men and continues to be a leading cause of cancer-related morbidity and mortality worldwide. Early detection and effective risk stratification are critical for improving clinical outcomes, guiding therapeutic decisions, and minimizing unnecessary interventions. While PSA testing has long been central to PCa screening, its limited specificity and prognostic reliability have spurred the development of more advanced diagnostic approaches. In response, platforms such as electrochemical sensors, lateral flow immunoassays, and nanoparticle-assisted assays have emerged, offering improved sensitivity and the ability to detect PSA isoforms or quantify PSA at ultralow concentrations [1,2,3,4].

Recent advancements at the intersection of nanotechnology, molecular diagnostics, and bioanalytical engineering have led to the development of next-generation biosensing platforms. Among these, QD-based technologies have attracted significant attention due to their unique optical and electronic properties, such as size-dependent fluorescence emission, high photostability, and their suitability for multiplexed signal detection [2,5,6,7]. These attributes position QDs as versatile components in diverse diagnostic applications, including immunoassays, fluorescence-based detection, electrochemical biosensing, and integration into point-of-care diagnostic tools.

Performance enhancements have also been achieved by combining QDs with other nanomaterials, such as graphene, metal–organic frameworks (MOFs), and carbon-based QDs. These hybrid platforms contribute to improved signal transduction, greater biocompatibility, and adaptability across various detection modalities [8,9,10,11]. In particular, the integration of hydrogel-based composites with QDs has shown promise in achieving real-time biosensing and smart, responsive diagnostics owing to their chemical stability, optical functionality, and responsiveness to physiological stimuli [12]. Such platforms are increasingly being explored not only in oncology but also in broader biomedical contexts, including wound care, wearable sensors, and regenerative medicine [13].

Notably, many of these biosensing systems have been optimized for use in complex biological matrices, including serum, urine, tissue extracts, and even live-cell environments, demonstrating strong potential for clinical translation [14,15,16,17]. Advances in peptide-functionalized nanocarriers, including QDs, have further enabled the targeted detection of PCa biomarkers with enhanced bioavailability and cellular specificity. This functionalization allows for improved biosensing and molecular imaging, potentially overcoming some of the limitations associated with off-target interactions and immune responses [18].

Considering the molecular and phenotypic heterogeneity of PCa, there is a growing demand for diagnostic strategies capable of multiplexed biomarker detection. Technologies that can concurrently identify different protein isoforms, gene fusions, or molecular signatures offer the potential to enhance diagnostic precision and support personalized oncology approaches [19,20,21,22,23]. Building on this, QDs have already demonstrated an impact on other cancer domains—most notably on triple-negative breast cancer—for precise imaging and targeted therapy, offering valuable cross-applications to PCa research [24].

Despite this progress, several challenges continue to hinder the clinical adoption of QD-based biosensing technologies. Key obstacles include concerns about cytotoxicity, limited long-term stability, complex regulatory approval processes, and the need for compatibility with the existing clinical infrastructure [14,15,16,17]. Furthermore, the integration of portable diagnostic systems, artificial intelligence-assisted image processing, and emerging theranostic features introduces new technical complexities that require a thorough evaluation of both hardware and software components [4,25,26,27]. The convergence of diagnostics and therapy, or nanotheranostics, is especially promising in this regard, as QD-enabled platforms increasingly support integrated photothermal and photodynamic therapies, offering dual-functionality for precision intervention while also introducing new engineering and translational challenges [28].

To ensure a comprehensive and focused review, a systematic selection strategy was employed. Studies were included if they (i) utilized QD-based technologies, (ii) specifically addressed PCa diagnostics or treatment, and (iii) were published in peer-reviewed journals between 2006 and 2025. Exclusion criteria ruled out studies unrelated to PCa, those lacking original experimental data, or publications in non-English languages. This review consolidates current advancements in QD-enabled biosensors for PCa detection, with a focus on the materials employed, platform architecture, signal transduction strategies, and biological interface compatibility. Special attention is given to how innovations in nanomaterials intersect with diagnostic performance and clinical feasibility, highlighting the potential of QD-based technologies to advance PCa diagnostics in the era of precision medicine.

## 2. Diagnostic Targets in PCa

### 2.1. PSA

PSA, a serine protease secreted by prostatic epithelial cells, has long been the cornerstone biomarker for PCa screening and disease monitoring. Although elevated PSA levels in serum are often indicative of malignancy, they can also result from benign conditions such as benign prostatic hyperplasia or prostatitis, leading to false positives and potential overtreatment [1,2,3,4]. To improve diagnostic specificity, clinical practice often distinguishes between total PSA (t-PSA), free PSA (f-PSA), and complexed PSA (c-PSA). In particular, the ratio of f-PSA to t-PSA is considered informative in the diagnostic gray zone of 4–10 ng/mL, where standard PSA testing is less conclusive [19,23,27].

### 2.2. Technological Advancements in PSA Detection

Recent developments in biosensor technology have significantly enhanced PSA detection by increasing sensitivity, reducing the assay time, and enabling multiplexed analysis. Electrochemical and aptamer-based sensors now offer real-time, label-free PSA detection at ultralow concentrations [9,10]. QD-based sensors—employing either fluorescence or electrochemical signal outputs—have achieved detection limits in the femtogram to picogram per milliliter range [8,9,29,30]. These advanced platforms also allow for the simultaneous detection of multiple PSA isoforms and related biomarkers, as summarized in Table 1.

### 2.3. Metabolic Biomarkers: Citrate, Sarcosine, and Zinc

The prostate gland’s unique metabolism offers a valuable context for biomarker discovery. In normal prostatic epithelial cells, high intracellular zinc levels inhibit mitochondrial aconitase activity, promoting the accumulation of citrate. However, in malignant cells, downregulation of the ZIP1 zinc transporter leads to reduced zinc uptake, reactivation of the Krebs cycle, and a subsequent decline in citrate levels [31,32,33,34]. Sarcosine, a metabolite derived from glycine, has also been proposed as a urinary biomarker associated with tumor progression, although its clinical utility remains under debate [35]. Moreover, intracellular zinc depletion itself reflects oncogenic metabolic reprogramming and may serve as a standalone diagnostic marker [36].

### 2.4. Genomic and RNA-Based Biomarkers

Advancements in genomic and transcriptomic profiling have identified several molecular markers with diagnostic relevance. PCA3 (prostate cancer antigen 3), a long non-coding RNA, is overexpressed in PCa and is most commonly detected in urine, though newer biosensors have enabled its detection in serum and cell lysates as well [37,38]. Another key marker is the transmembrane protease serine 2—v-ets erythroblastosis virus E26 oncogene homolog (*TMPRSS2–ERG*) gene fusion that is present in nearly 50% of PCa cases. This fusion is frequently found alongside phosphatase and TENsin homolog (*PTEN*) deletions and is associated with more aggressive disease phenotypes [15,20,22].

### 2.5. Protein Biomarkers Beyond PSA

In addition to PSA, several other protein biomarkers provide diagnostic and prognostic value. Prostate-specific membrane antigen (PSMA), a transmembrane glycoprotein, is significantly overexpressed in PCa and serves both as a diagnostic marker and a therapeutic target. PSMA-targeted agents, often conjugated with fluorophores or radionuclides, enable the visualization of micro metastases and tumor margins [39,40,41]. Other proteins, such as prostate stem cell antigen (PSCA) and alpha-methyl acyl-CoA racemase (*AMACR*), show correlations with the tumor grade and histological subtype [14,42]. Diagnostic panels frequently combine these markers with PSA, and sometimes carcinoembryonic antigen (CEA), to enhance specificity [7]. These protein biomarkers are incorporated in Table 1 as part of multi-analyte diagnostic frameworks.

### 2.6. Emerging Functional and Imaging-Based Biomarkers

Recent innovations in molecular imaging and diagnostics have enabled higher-resolution characterization of PCa. Ligands targeting PSMA or folate receptors and conjugated with QDs or near-infrared dyes allow for the real-time visualization of the tumor margins and metastatic lesions [39,43]. At the cellular level, the co-detection of androgen receptor (AR) and PSA expression can yield insights into hormonal signaling and therapeutic resistance [44]. Furthermore, markers of the epithelial–mesenchymal transition (EMT), including E-cadherin, N-cadherin, Vimentin, and receptor activator of nuclear factor kappa-B ligand (RANKL), are being explored for their ability to assess metastatic potential and disease progression [45,46]. These biomarkers are classified in Table 1.

**Table 1:** Multiplexed PCa biomarker detection using QD-based technologies: targets, methods, and diagnostic value.

Biomarker Class/Detection Target	Mechanistic Insights and Representative Approaches	Clinical Utility and Translational Implications	Supporting Studies
PSA as the Core Diagnostic Biomarker	PSA is the most prevalent target in PCa diagnostics. It is detected as total, free, or complex forms using electrochemical, surface plasmon resonance (SPR), aptamer-based, and fluorescent technologies. The detection limits range from fg/mL to attomolar.	Enables early-stage detection, longitudinal monitoring, and quantitative assessments of PCa. Foundational for both screening and follow-up in clinical settings.	[1,3,29,30,47,48]
PSA-Based Multiplex Detection	PSA is combined with markers like CEA, ATP, AR or detected in isoforms (f-PSA, c-PSA) using QD-labeled arrays, dual-color probes, and microfluidic formats.	Increases diagnostic specificity and reduces false positives. Useful for risk stratification and differentiation between benign and malignant conditions.	[7,19,21,23,27,44,49]
Non-PSA Biomarkers (Single Target)	Includes metabolic (citrate and sarcosine), RNA (PCA3), protein (DNER and Akt-1), and hormone pathway (IGF-1) markers. Technologies include electrochemical sensors and fluorescence-based quantitation.	Provide complementary biomarkers for early detection, disease progression monitoring, or assessing the therapeutic response. Often used alongside PSA.	[26,32,33,34,35,37,50,51]
Multiplexed Non-PSA Biomarkers	Simultaneous detection of multiple non-PSA markers (e.g., *ERG*, *PTEN*, EMT-related proteins, and E-cadherin) using QDs, IHC, or nanoprobes. Applied to tissue sections or cell-level assays.	Enable tumor subtyping and molecular classification. Support personalized treatment planning, a prognostic estimation, and recurrence predictions.	[15,20,45,46,52]
PSMA-Based Imaging and Theranostics	PSMA-targeted QDs and near-infrared (NIR) imaging agents allow the selective visualization of prostate tumors and micro-metastases. Applied in deep tissue or in vivo models.	Facilitate image-guided biopsy and surgical planning. Potential for targeted therapy and monitoring of advanced-stage disease.	[39,40,41]
Therapy/Imaging Without Multiplexing	Use of nanocarriers, QDs, or aggregation-induced emission dots (AIE-dots) for drug delivery, phototherapy [photothermal therapy (PTT)/photodynamic therapy (PDT)], or apoptosis induction in PCa cells.	Supports the preclinical development of targeted therapies and non-invasive imaging. Demonstrates the feasibility of nanoparticle-guided treatment strategies.	[16,17,43,53,54,55,56,57]

## 3. Clinical Relevance and Multiplexing Capability in PCa Diagnostics

The integration of nanotechnology into biosensing platforms has significantly enhanced the landscape of PCa diagnostics. QD-based systems, particularly those optimized for multiplexing and signal amplification, have enabled greater sensitivity, broader biomarker coverage, and real-time diagnostic functionality. These innovations directly address longstanding limitations in early detection, assay specificity, individualized risk stratification, thereby expanding the clinical relevance and utility of biosensors.

### 3.1. Clinical Relevance via Ultrasensitive PSA Detection

PSA remains the most widely utilized biomarker for PCa screening and disease monitoring. However, conventional assays often lack the sensitivity required to detect PSA at very low concentrations, especially in early-stage or borderline cases. QD-based biosensors have addressed this limitation with remarkable improvements in detection sensitivity. For example, aptamer-based sensors using *gold–carbon QD* (*Au/CQD*) hybrids have achieved detection thresholds as low as 2 fg/mL [30], while sensors incorporating *GQDs* have demonstrated limits of 0.66 pM with excellent reproducibility [9]. Even greater sensitivity was observed using *sulfur-doped GQDs* combined with gold nano stars, achieving 9.7 aM in buffer and 26 fg/mL in serum [29]. Electrochemical and electrochemiluminescence (ECL) platforms have also delivered high-performance results, with one *GQD–TiO_2_* nanotube system reaching a 1 fg/mL limit in serum [58]. These QD-enhanced biosensors have shown strong agreement with conventional diagnostic assays such as ELISAs and chemiluminescent immunoassays, reinforcing their readiness for clinical integration [3,59,60].

### 3.2. Multiplexing: Expanding the Diagnostic Breadth and Depth

Given the molecular heterogeneity of PCa, single-marker diagnostics are often insufficient for comprehensive disease characterization. QD-based biosensors designed for multiplexed detection improve diagnostic accuracy by simultaneously quantifying multiple PSA isoforms and incorporating additional protein or genomic biomarkers. For instance, dual-color magnetic QD nanobeads have been used to concurrently detect free PSA (f-PSA) and complexed PSA (c-PSA), achieving detection limits of 0.009 ng/mL and 0.087 ng/mL, respectively, both well within clinically relevant ranges [27]. Suspension bead arrays and lab-on-a-bead microarrays have enabled high-throughput measurements of total and free PSA levels with high specificity [19,23]. Multiplexed detection has also been extended to include markers such as carcinoembryonic antigen (CEA) and adenosine triphosphate (ATP), with paper-based and fluorescent assays designed for decentralized and resource-limited settings [7,49,61].

QD biosensors have also achieved high sensitivity in genomic diagnostics. Multicolor QD assays have been used to detect *TMPRSS2–ERG* fusion genes and PTEN deletions at concentrations as low as 1 fmol in urine and cell lysates, with performance comparable to RT-PCR [15,20,22]. Spatial biomarker profiling has been accomplished using QD-labeled nanoarrays and imaging assays for proteins like androgen receptor (AR), E-cadherin, and RANKL, further supporting multiplexed diagnostics and tumor microenvironment analyses [44,45,62].

### 3.3. Translational Readiness and Clinical Integration

To achieve widespread clinical adoption, biosensors must combine analytical performance with ease of use, reproducibility, and robustness in diverse sample environments. QD-based platforms are progressing in this direction through miniaturized and cost-effective formats tailored for clinical workflows and point-of-care applications. An electrochemical sensor incorporating *Au–GQDs* on indium tin oxide (*ITO*) electrodes was able to detect PSA in under five minutes, while maintaining signal stability in human serum [38]. Smartphone-integrated lateral flow assays using QD-embedded silica nanoparticles delivered accurate PSA measurements and maintained signal fidelity over a 10-day period [4]. The incorporation of hydrophilic polymers and anti-fouling coatings has further enhanced sensor stability and reduced matrix-related signal interference [10,47,63].

Some QD-based platforms have demonstrated dual functionality for both diagnosis and therapy, enabling theranostic workflows. For example, *polydopamine–folate carbon dots* (*PFCDs*) and aggregation-induced emission (AIE) nanodots have been used to detect PSA and simultaneously deliver photothermal treatment to PCa cells [46,61]. Additionally, QD fluorescence in situ hybridization (QD-ISH) and multiplex immunohistochemistry (QD-IHC) have been adapted for use with formalin-fixed, paraffin-embedded (FFPE) tissue, allowing the simultaneous high-resolution visualization of both protein and nucleic acid biomarkers [15,20].

**Scheme 1:**
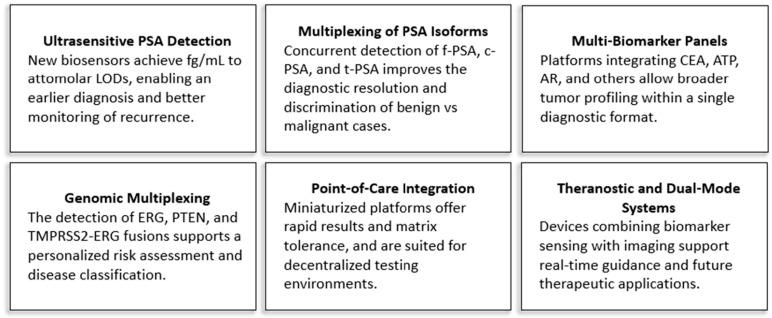
Summary of clinically relevant innovations and multiplexing strategies in PCa biosensing. The figure categorizes references by diagnostic capability, including ultrasensitive PSA detection, multiplexing of PSA isoforms, multi-biomarker panels, genomic profiling, point-of-care integration, and theranostic dual-mode systems. References—Ultrasensitive PSA Detection: [3,4,9,29,30,58,59,60,64]; Multiplexing of PSA Isoforms: [19,21,23,27]; Multi-Biomarker Panels: [7,44,45,49]; Genomic Multiplexing: [15,20,22,62]; Point-of-Care Integration: [10,38,47,63,65]; Theranostic and Dual-Mode Systems: [46,61].

QD-based biosensors have evolved from laboratory tools into clinically relevant diagnostic platforms. By addressing both the sensitivity gap and the multiplexing limitations of conventional assays, QD-based platforms substantially improve the diagnostic accuracy and clinical confidence. Their ability to achieve ultralow detection limits, support a multiplexed analysis, and perform reliably across complex biological matrices highlights their translational maturity. These systems now extend beyond PSA measurements to enable comprehensive molecular and genetic profiling, offering critical support for early detection, disease monitoring, and precision therapeutic planning. Their adaptability across imaging, point-of-care, and even therapeutic contexts positions QD-enabled biosensing at the forefront of next-generation diagnostics in PCa care [3,4,7,19,21,59]. Scheme 1 presents a synthesized overview of key diagnostic innovations, target biomarkers, and system capabilities supporting the shift toward precision PCa care.

## 4. Detection Platforms in PCa Diagnostics

### 4.1. QD-Based Platforms as Central Frameworks

QDs have emerged as core components in PCa diagnostics owing to their size-tunable fluorescence, narrow emission spectra, and strong photostability. These attributes have enabled their integration into highly sensitive, miniaturized, and multiplexed biosensor systems.

Semiconductor QDs such as cadmium selenide/zinc sulfide (*CdSe@ZnS*) and cadmium telluride (*CdTe*) have been incorporated into lateral flow immunoassays (LFIAs) and paper-based diagnostic devices, significantly improving detection sensitivity. For example, silica-coated *CdSe@ZnS* QDs synthesized via reverse microemulsion have demonstrated minimal nonspecific binding and a quantum yield of 36.5%, enabling effective PSA detection with LFIA formats [2].

Carbon-based QDs, including carbon QDs (*CQDs*) and graphene QDs (*GQDs*), provide enhanced biocompatibility and low toxicity, broadening their applicability in diagnostic systems. These materials have been successfully employed in label-free electrochemical PSA sensors [10] and in optical detection platforms targeting sarcosine [35]. While CQDs are commonly used in electrochemical applications, their potential in Förster resonance energy transfer (FRET)-based diagnostics remains largely untapped and represents a promising avenue for future exploration.

### 4.2. Electrochemical and Electro-Chemiluminescent Detection Platforms

Electrochemical biosensors are widely utilized in PCa diagnostics due to their high sensitivity, rapid detection, and compatibility with portable devices. When QDs are combined with conductive nanomaterials, such as gold nanoparticles (*AuNPs*), graphene oxide, and carbon composites, they facilitate enhanced electron transfer and signal amplification.

For instance, aptamer-based electrochemical sensors using reduced graphene oxide–*AuNP* composites and *CQDs* have achieved PSA detection limits as low as 3 pg/mL [10]. Platforms using *Au/CQD* hybrids paired with methylene blue as a redox probe have demonstrated even greater sensitivity, reaching detection limits of 2 fg/mL [30].

Electro-chemiluminescence (ECL) platforms build on electrochemical systems by amplifying optical outputs. Recent systems incorporating graphitic carbon nitride QDs (*g-C_3_N_4_ QDs*) with two-dimensional transition metal carbide and nitride (*MXene*) nanosheets and gold manganese dioxide (*Au@MnO_2_*) nanostructures have achieved femtogram-level sensitivity through dual quenching effects [66]. These hybrid platforms represent robust and reproducible alternatives to traditional chemiluminescence methods.

### 4.3. Hybrid Nanomaterials for Signal Amplification

Hybrid nanomaterials significantly enhance the functional capabilities of QD-based biosensors by merging the optical properties of QDs with the electrical, catalytic, or structural advantages of complementary materials. For example, immunosensors incorporating *poly*(*thionine*), GQDs, and activated carbon have achieved dual-range PSA detection with limits as low as 0.005 ng/mL [11]. MOF–QD hybrids have also been employed in enzyme-free sandwich assays, demonstrating excellent selectivity and stability in serum environments [67]. Further developments include aptamer-functionalized QDs conjugated with *cobalt phthalocyanines*, which offer robust electrochemical stability and picomolar-level sensitivity [9,68]. These hybrid systems support multimodal detection formats, enhancing adaptability across diverse diagnostic applications.

### 4.4. Immunoassay Formats and Multiplexing Capabilities

QD-based technologies have expanded the functional scope of traditional immunoassays, enabling enhanced sensitivity and multiplexed biomarker detection in both laboratory and point-of-care settings.

QD-enhanced lateral flow devices support dual-analyte detection—for example, PSA and carcinoembryonic antigen (CEA)—with high signal-to-noise ratios and minimal cross-reactivity [7]. Suspension microarrays using dual-color QD nanobeads facilitate the simultaneous detection of free PSA and complexed PSA, with sensitivity levels comparable to the standard enzyme-linked immunosorbent assay (ELISA) [23,27].

In tissue diagnostics, QD-based fluorescence in situ hybridization (QD-FISH) and multiplexed immunohistochemistry enable long-term, high-resolution imaging of key biomarkers such as ETS-related gene (*ERG*), phosphatase and TENsin homolog (*PTEN*), prostate stem cell antigen (PSCA), and androgen receptor (AR) [20,42,44,69]. These imaging tools are especially valuable for spatial biomarker localization during tumor grading and staging.

### 4.5. Theranostic and Imaging-Oriented Platforms

QDs are also integral to emerging theranostic systems that combine diagnostic imaging with therapeutic delivery. Folic acid (*FA*)-conjugated QDs have demonstrated the selective targeting of *FA* receptor-overexpressing PCa cells, supporting both visualization and drug delivery functions [43,70,71].

Near-infrared (NIR) QDs such as QD800 and silver indium selenide/zinc sulfide (*AgInSe/ZnS*) enable the real-time imaging of tumor margins and deep tissue metastases [17,39]. These probes are valued for their rapid clearance and high contrast, making them suitable for intraoperative imaging and noninvasive diagnostics.

Therapeutically, QD-loaded liposomes and aggregation-induced emission (AIE)-dot vesicles have been used to achieve synergistic photodynamic and photothermal effects on prostate tumor models [56,72]. These multifunctional platforms align with precision oncology by facilitating localized, image-guided treatments.

### 4.6. Miniaturized and Disposable Platforms for Point-of-Care Applications

The demand for decentralized diagnostics has spurred the development of compact, cost-effective platforms with strong analytical performance. In this area, QD-based technologies show particular promise. Paper-based immune devices incorporating cadmium telluride (*CdTe*) QDs have demonstrated robust multiplexed detection with recovery rates exceeding 100% in serum samples [7]. Screen-printed electrodes modified with molybdenum disulfide (*MoS_2_*) QDs have reached PSA detection thresholds as low as 0.01 pg/mL and have shown strong performance in spiked serum samples, making them suitable for field deployment [73].

QD-microbead arrays further enable high-throughput biomarker detection using portable fluorescence readers or smartphone-integrated systems [19,23]. These developments bring QD-enabled diagnostics closer to home-based or remote screening applications. A comprehensive summary of these detection platforms, including the nanomaterial types, signal transduction mechanisms, and diagnostic applications, is presented in Table 2. Collectively, these advances highlight the central roles of QDs as both signal amplifiers and modular elements in the design of next-generation, personalized PCa diagnostic tools.

## 5. Signal Transduction Mechanisms in Nano Biosensing Platforms

Signal transduction lies at the heart of nano biosensing, converting molecular recognition events into measurable outputs. In PCa diagnostics, the choice of signal transduction mechanism is critical, as it directly impacts sensitivity, scalability, and clinical applicability. Thanks to their unique optical and electronic properties, QDs support a wide range of transduction modes, including electrochemical, optical, and hybrid strategies. This section outlines the primary signal conversion mechanisms utilized in QD-based diagnostic platforms for PCa.

### 5.1. Electrochemical Transduction

Electrochemical transduction remains a foundational approach in QD-enabled biosensors due to its high sensitivity, rapid response time, and compatibility with compact instrumentation. QDs enhance these systems by facilitating electron transfer and enabling effective surface functionalization, thereby improving the detection resolution.

For instance, cobalt *phthalocyanine*-functionalized QDs immobilized on aptamer-modified glassy carbon electrodes enabled PSA detection at concentrations as low as 0.66 pM, benefiting from combined electrocatalytic activity and π–π stacking interactions [9]. *Cu-doped carbon QDs* embedded within reduced graphene oxide–gold nanocomposites achieved detection limits of 3 pg/mL using square wave and cyclic voltammetry techniques [10]. Similarly, nitrogen-doped graphene QDs (*NGQDs*) enhanced the signal strength in impedance-based platforms, reaching a PSA detection threshold of 1.54 pM in serum [68]. Some systems also employ dual-mode strategies—combining differential pulse voltammetry (DPV) and electrochemical impedance spectroscopy (EIS)—to increase both sensitivity and diagnostic confidence [77].

### 5.2. Fluorescence-Based Transduction

Fluorescence transduction exploits the inherent brightness and photostability of QDs, enabling the real-time and highly sensitive detection of PCa biomarkers. These systems span from basic immunofluorescence assays to advanced Förster resonance energy transfer (FRET) configurations.

Simple implementations include QD–antibody conjugates used in lateral flow immunoassays and suspension microarrays to detect PSA isoforms, offering high signal clarity and low background interference [2,23]. Advanced designs, such as FRET-based systems, use terbium (*Tb*)*-*labeled antibodies to transfer energy to QDs (e.g., QD705), allowing multiplexed PSA detection with sensitivity down to 2 nM [21]. “Turn-on” systems, such as those using *GQDs* and silver (*GQDs@Ag*) core–shell nanocrystals, restore fluorescence upon Ag shell etching and have demonstrated pg/mL-level PSA detection in serum with minimal interference [8].

Fluorescence-based QDs are also crucial for in vivo imaging applications. For example, MoS_2_ QDs facilitate two-photon luminescence imaging of prostate-specific membrane antigen (PSMA)-positive tumors at subcellular resolution [40], while copper indium sulfide (*CuInS_2_*) QDs conjugated with mucin 1 (*MUC1*) aptamers enable dual imaging and chemotherapeutic monitoring in PCa cells [16].

### 5.3. Electrochemiluminescence (ECL)

ECL platforms combine electrochemical activation with light emission, offering ultra-sensitive detection and high signal specificity. QDs serve as efficient ECL emitters due to their strong, stable luminescence under electrical excitation.

A ternary ECL system utilizing graphitic carbon nitride QDs (*g-CNQDs*), silver nanoparticles on a titanium carbide (*Ag@Ti_3_C_2_*) *MXene* platform, and *Au@MnO_2_* nanostructures achieved dual quenching effects and a PSA detection limit of 6.9 fg/mL [66]. Another platform based on *TiO_2_* nanotube arrays enhanced with *GQDs* and *CdTe* magnetic particles pushed the sensitivity even further to 1 fg/mL in serum samples [58]. These systems are especially advantageous for complex biological matrices, offering high signal-to-noise ratios and minimal background interference.

### 5.4. Photoelectrochemical (PEC) and Photoluminescence (PL) Mechanisms

PEC and PL mechanisms leverage the light-responsive behavior of QDs for biomolecular detection. In PEC sensors, a photocurrent is generated upon light exposure, with changes in the signal corresponding to the analyte presence.

For example, cadmium sulfide (*CdS*) QDs integrated into titanium dioxide (*TiO_2_*) functionalized with gold nanoparticles (*TiO_2_–AuNP*)*–graphene* composites utilize exciton–plasmon coupling. In these systems, aptamer displacement upon PSA binding restores the photocurrent, enabling PSA detection at 0.52 pg/mL [6]. Similarly, a *CdS/MoS_2_* heterojunction sensor employed immunoreaction-induced Cu^2+^ generation to inhibit QD formation, achieving a detection limit of 0.29 pg/mL [64].

Photoluminescent approaches, on the other hand, rely on emission modulation. *CuInS_2_* QDs functionalized with *MUC1* aptamers supported both imaging and therapeutic monitoring by tracking fluorescence during daunorubicin release, with sensitivity down to 19 nM [16].

### 5.5. Hybrid Signal Transduction Platforms

Hybrid transduction strategies combine multiple signal modalities—typically optical and electrochemical—to enhance diagnostic robustness and allow multimodal data interpretation. These platforms are especially valuable for multiplexed assays and portable diagnostic systems.

An example is sulfur-doped carbon dots conjugated to anti-PSA antibodies, which support both electrochemical immunoassay (ECIA) and fluorescence immunoassay (FIA) modes. This dual-mode sensor achieved a detection limit of 38 pg/mL and was compatible with smartphone-based readers, offering potential for point-of-care use [61]. Another hybrid system employs dual-color QD nanobeads in magnetic lateral flow assays for the simultaneous detection of free and complexed PSA. The emission of distinguishable wavelengths supports an accurate PSA evaluation within the diagnostic gray zone [27].

By integrating optical and electrochemical signals, hybrid biosensors reduce false positives, improve diagnostic confidence, and enable data-rich analysis—qualities that are particularly important in high-risk conditions like PCa.

## 6. Nanomaterial Composition in Biomedical Sensing and Diagnostics

The composition of nanomaterials plays a pivotal role in shaping biosensor performance, particularly in PCa diagnostics, where parameters such as sensitivity, biocompatibility, and signal stability are crucial. Nanomaterial QDs, carbon-based structures, and hybrid composites contribute to enhanced transduction efficiency, spectral resolution, and target specificity. By optimizing the composition and applying precise surface engineering, these materials facilitate the development of next-generation biosensors with significant clinical potential. Their functional roles are detailed in Table 3.

### 6.1. QDs: Foundational Nanomaterials in Biosensing

QDs are key building blocks in biosensor platforms owing to their size-tunable emission wavelengths, sharp spectral profiles, and high quantum yields. Cadmium-based QDs, such as *CdTe*, *CdSe*, and *CdS*, are widely employed in PCa diagnostics for their reliable and bright fluorescence. For example, silica-coated *CdSe@ZnS* QDs achieve long-term colloidal stability and quantum yields of approximately 36.5%, enabling their effective use in PSA detection via lateral flow assays [2]. However, toxicity concerns surrounding cadmium have accelerated the shift toward alternative, biocompatible QD compositions. Manganese-doped zinc selenide (*Mn:ZnSe*) QDs have been used for alpha-methyl acyl-CoA racemase (*AMACR*)-targeted imaging with minimal cytotoxic effects [14], while *CuInS_2_* QDs conjugated with MUC1 aptamers offer tumor-specific imaging and therapeutic tracking in PCa cells [16]. *AgInSe/ZnS* QDs, which emit in the near-infrared (NIR) range, combine low toxicity with excellent imaging capabilities for cancer and microbial targets [17]. These cadmium-free formulations retain desirable photophysical characteristics while improving clinical safety profiles.

### 6.2. Carbon-Based QDs: Safe and Functional Alternatives

*CQDs* and *GQDs* are increasingly favored in biomedical diagnostics due to their superior biocompatibility, low toxicity, and structural versatility. Their ease of functionalization and aqueous solubility make them suitable for a wide range of biosensing applications.

*GQDs* integrated with gold nanostructures have enabled electrochemical multiplexing platforms for PSA detection, benefiting from improved electrocatalytic activity [38]. *CQDs* synthesized from green sources, such as *sulfur-doped* variants derived from onion extract, have demonstrated PSA detection at concentrations as low as 38 pg/mL using dual-mode fluorescence and electrochemical assays [61]. Heteroatom doping, including nitrogen and boron, enhances both charge transport and luminescence. For instance, *nitrogen-doped CQDs* enable citrate detection at 2.2 × 10^−8^ M in complex matrices [33], while *boron-doped carbon dots* are under investigation for boron neutron capture therapy and immune modulation in cancer treatment [57].

### 6.3. Hybrid Nanocomposites: Multifunctional Signal Amplification

Hybrid nanocomposites combine QDs with metallic, polymeric, or two-dimensional (2D) materials to construct multifunctional architectures that amplify the signal response and improve material stability.

*CdS* QDs embedded within *AuNP*-coated graphene structures have enabled signal amplification through exciton–plasmon coupling, supporting label-free photoelectrochemical (PEC) PSA detection at femtomolar sensitivity [6]. Turn-on fluorescence systems using *GQDs@Ag* core–shell structures have reached PSA detection limits as low as 0.3 pg/mL, where Ag shell etching activates the fluorescence signal [8].

Additional hybrid systems utilize materials like *MoS_2_* and *g-C_3_N_4_* to enhance light absorption and electron transfer. A molybdenum disulfide/graphitic carbon nitride/gold (*MoS_2_/g-C_3_N_4_/Au*) composite demonstrated reproducible PSA detection in serum samples [1], while a graphitic carbon nitride–palladium (*g-C_3_N_4_–Pd*) nanohybrid supported aptamer-based fluorescence sensing with a detection limit of 4.2 pg/mL [78]. These designs promote dual-mode detection and are adaptable for multi-marker diagnostic assays.

### 6.4. Surface Functionalization with Polymers and Ligands

Surface functionalization is critical for rendering nanomaterials biologically active, stable, and target-specific. Functional polymers like *poly*(*thionine*) and *polydopamine* enhance electrochemical performance, biocompatibility, and target-binding efficiency.

For example, *poly*(*thionine*)-coated GQDs embedded in carbon matrices derived from marigold flower biomass enabled dual-range PSA detection with a detection limit of 0.005 ng/mL [11]. *Polydopamine*-coated folate carbon dots were used to selectively target PSMA-expressing PCa cells for combined imaging and photothermal therapy [41].

Ligand modifications further increase specificity. Folic acid and β-cyclodextrin conjugated to *CdTe* QDs have been used for targeted imaging and drug delivery in prostate and lung cancer models [70]. Aptamer functionalization—targeting markers such as PSA or *MUC1*—enhances molecular recognition in both fluorescence and electrochemical formats [16,61]. These surface modifications are key to ensuring diagnostic accuracy and in vivo compatibility.

### 6.5. Multiplexing and Spectral Engineering via Compositional Control

Controlling the QD composition and emission profiles allows for the precise spectral tuning and multiplexed detection of multiple biomarkers. For example, QDs with emissions at 605, 655, and 705 nm have been deployed in FRET-based microarrays to quantify PSA isoforms concurrently, with QD705 achieving the lowest detection limit of 2 nM [21].

Dual-color QD nanobeads emitting at green and red wavelengths facilitate the simultaneous detection of free and complexed PSA, supporting a differential diagnosis of ambiguous clinical cases [23]. Spectral tuning via elemental doping (e.g., Mn^2+^, S, and B) and shell passivation further enhances the fluorescence stability and target specificity.

Mn-doped ZnSe QDs conjugated with anti-AMACR antibodies demonstrated selective tumor cell labeling with minimal background interference [14], while Mn-doped ZnS QDs functionalized with dihydrolipoic acid supported phosphorescent PSA detection at 17 pg/mL, even in complex biological matrices [80]. These engineered platforms enable an early-stage diagnosis and personalized treatment planning through robust and multiplexed signal outputs.

A detailed outline of these innovations is presented in Table 3, which categorizes the nanomaterial composition, transduction strategy, and clinical utility in PCa diagnostics.

**Table 3:** Performance and advantages of QD nanocomposites in PCa diagnostics: material-specific innovations.

QD Class and Integration Strategy	Key Performance Metrics and Representative Examples	Advantages and Biomedical Application Insights	Representative References
Carbon-Based QDs (*CQDs*, *GQDs*, and *g-C_3_N_4_ QDs*)	Achieve attomolar to low pg/mL LODs; widely used in ECL, fluorescence, and electrochemical sensors; examples include *GQDs@Ag* (0.3 pg/mL), *S-GQD@AuNS* (9.7 aM), and *GQD/TiO_2_* (1 fg/mL)	Abundant functional groups allow easy biofunctionalization; suitable for dual-mode and miniaturized biosensors	[8,29,47,58,60]
Gold-Enhanced QD Systems	*AuNPs* or *Au nanorods* integrated with QDs yield amplified signals and ultra-low LODs (fg–aM), e.g., *Au/CQD* (2 fg/mL) and *ZnCdHgSe* QDs + *AuNRs* (0.1 pg/mL)	Highly suitable for point-of-care diagnostics; a stable plasmonic response enhances optical and electrochemical outputs	[29,30,38,75]
*MoS_2_-*Based Composites	MoS_2_ nanosheets or zero-D QDs in hybrids (e.g., CdS/MoS_2_ and *MoS_2_@g-C_3_N_4_@AuNPs*) reach sub-pg/mL detection; validated in serum	High electrocatalytic surface area and conductivity; supports robust sensor platforms under biological conditions	[1,64,73]
*Cd-Based QDs* (*CdS*, *CdTe*, and *CdSe*)	Cd-based QDs show improved LODs in hybrid systems; *CdS/MoS_2_* (0.29 pg/mL) and *CdSe/CdTe* in FRET (15 ng/L)	Strong photoluminescence for imaging; require surface passivation or composites for optimal biosensing	[64,65,72,81]
QD Nanobeads and Multiplex Labeling	Nanobeads with QDs enable dual PSA isoform detection, e.g., red/green magnetic QDs (0.009 ng/mL f-PSA); validated against ELISA	Effective in simultaneous multi-analyte assays; compatible with rapid, low-volume clinical screening	[23,27,44]
QD-Based Imaging and Immunohistochemistry	QDs applied in IHC/FISH outperform dyes in stability and resolution, e.g., QD-PSCA (r = 0.732 with tumor grade) and QD-FISH (100% IHC concordance)	Enable long-term fluorescent tracking in tissues; enhance diagnostic precision in fixed biopsy samples	[15,20,42,50,69]

## 7. Manufacturing Methods of QD-Based Platforms for PCa Applications

The performance, reproducibility, and scalability of QD-based technologies depend critically on the methods used for QD synthesis and integration. This section reviews key fabrication strategies, such as hydrothermal processing, microwave-assisted synthesis, green chemistry, and surface engineering, that underpin the development of reliable, biocompatible, and clinically translatable QD-enabled platforms.

### 7.1. Hydrothermal and Microwave-Assisted Synthesis

Hydrothermal synthesis is one of the most prevalent techniques for producing carbon-based and doped QDs, and is valued for its operational simplicity, scalability, and use of low-toxicity precursors. Performed in sealed autoclaves at elevated temperatures and pressures, this method allows for controlled doping, particle size regulation, and surface passivation in a single step.

For example, sulfur-doped carbon dots synthesized hydrothermally from an onion extract were incorporated into dual-mode biosensors for PSA detection. These sensors leveraged both fluorescence and electrochemical outputs and demonstrated excellent biocompatibility and stability [61]. Similarly, microwave-assisted synthesis offers a rapid and energy-efficient alternative, yielding uniform nanoparticles with consistent optical properties. *Mn-doped ZnSe* QDs fabricated using this method served as photostable probes for selectively labeling *AMACR*-expressing PCa cells without off-target fluorescence [14]. Both methods support the reproducible, large-scale production of QDs suitable for integration into biosensing devices.

### 7.2. Green Synthesis of QDs

Green synthesis techniques are gaining favor in biomedical nanotechnology due to their environmentally responsible processes and inherent safety profiles. Common green approaches utilize plant extracts, fruit-derived polysaccharides, or microbial agents as carbon sources and natural reducing agents, eliminating the need for harsh chemicals [82,83]. These strategies are well aligned with global regulatory trends promoting safe and sustainable nanomaterial development.

**Figure 1:**
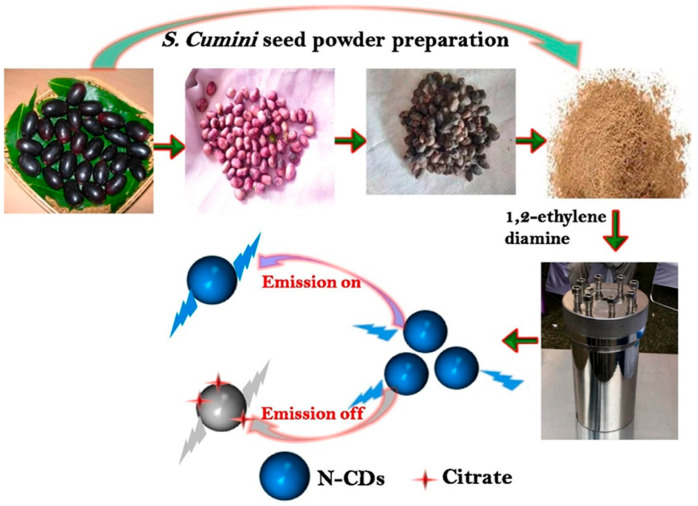
Schematic representation of the preparation of N-CDs form *S. Cumini* seeds; *Syzygium Cumini* seeds and 1,2-ethylenediamine were used as carbon and nitrogen sources for the biogenic synthesis of N-CDs, respectively, and its sensing strategy with citrate is shown. Adopted with permission [34].

A notable example is the synthesis of nitrogen-doped carbon dots (*N-CDs*) using *Syzygium Cumini* seed extract. The seeds are ground into a powder and combined with *1*,*2-ethylenediamine* as a nitrogen source (Figure 1). The resulting *N-CDs* exhibit strong fluorescence and were successfully used as probes for detecting citrate in urine and PCa cells, achieving a detection limit of 3.5 nM [34]. Their sensing mechanism involves a distinct “emission on/off” fluorescence changes upon citrate binding, enabling real-time, sensitive bioimaging for diagnostic applications.

Numerous green synthesis methods for QDs have been explored using a wide range of natural carbon sources. *CQDs* and *GQDs* have been synthesized from unconventional and renewable materials such as bread slices [83], *Illicium verum* (star anise) [84], citrus peel extract [85], and *Chenopodium album* (bathua leaves) [86]. These are typically produced via low-temperature hydrothermal or pyrolysis processes that minimize waste and avoid toxic solvents.

Green synthesis has also been extended to metal and semiconductor QDs. For instance, ginger and cardamom extracts have been used to produce cadmium sulfide (*CdS*) QDs with anticancer and antimicrobial activity [87]. Silver QDs have been derived from *Citrus limetta* peel [88], while tea leaf extract (*Camellia sinensis*) has served as a green precursor for *CdS* QDs with antibacterial and imaging capabilities [89].

These naturally synthesized QDs exhibit desirable biomedical properties, including a sub-10 nm size, tunable fluorescence, high water dispersibility, and low cytotoxicity. Green-synthesized *CQDs* and *GQDs* have shown efficacy in targeted bioimaging, photodynamic therapy, and drug delivery across multiple cancer types, such as colon cancer [83,90], breast cancer [84,91], lung cancer [89], PCa [11,91], neuroblastoma [86,92], and melanoma [93].

Furthermore, green QDs have been integrated into smart, tumor-responsive nanocarriers. For example, carbon dots from *Cinchona pubescens* exhibited high carbo-platin loading (~100 μg/mg) and pH-triggered, diffusion-controlled drug release [94]. Another platform involved embedding *CQDs* in a *carboxymethyl cellulose/agarose* hydrogel matrix to deliver quercetin. This nanocarrier system displayed enhanced drug release at an acidic pH (5.4) mimicking the tumor environment, and effectively induced apoptosis in A549 lung cancer cells [95]. However, it is important to note that while green synthesis reduces the use of hazardous reagents, the long-term biosafety and environmental impacts of green-synthesized metal-based QDs, such as CdS produced from plant extracts, remain under active investigation and require further toxicological validation.

### 7.3. Reverse Microemulsion and Encapsulation Techniques

Reverse microemulsion synthesis is a highly effective approach for producing QDs with precise control over the particle size, morphology, and core–shell architecture. This technique enables the fabrication of well-defined nanostructures, such as *CdSe@ZnS* QDs, with consistent emission profiles and enhanced photostability [2]. These controlled features make reverse microemulsion-derived QDs particularly suitable for advanced bioimaging and theranostic applications.

**Figure 2:**
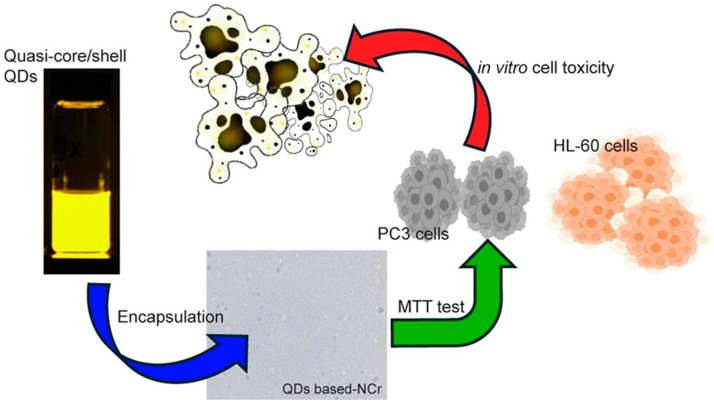
An illustration showing semiconductor nanocrystals, also known as QDs, that are particularly promising components of drug carrier systems due to their small sizes and robust photoluminescence properties. Adopted with permission from [72].

A further enhancement of QD functionality is achieved through encapsulation methods, which improve colloidal stability, enable drug co-loading, and facilitate targeted therapeutic delivery. As shown in Figure 2, an advanced multifunctional nanocarrier was developed by encapsulating cadmium, zinc, selenium, and sulfur (*CdZnSeS*) QDs within *alginate-*based nanocarriers co-loaded with the anticancer agents *betulinic acid* and *ceranib-2.* This engineered QD-based nanocarrier (QDs-NCr) exhibited enhanced cytotoxicity against PC-3 PCa cells [72]. The therapeutic performance of the QDs-NCr system was validated using MTT cell viability assays, which demonstrated significant in vitro toxicity not only in PC-3 PCa cells but also in HL-60 leukemia cells. Encapsulation plays a dual role: it preserves the intrinsic photoluminescence of QDs while providing a platform for targeted, synergistic drug delivery.

### 7.4. Electrochemical Deposition and in Situ Assembly

Electrochemical deposition is a powerful technique for fabricating QD-based sensing interfaces, offering precise control over the film thickness, uniformity, and spatial localization. These attributes are critical for achieving reproducible and high-performance biosensors.

For instance, a PCA3-targeted biosensor employed Au–graphene QDs electrochemically deposited onto indium tin oxide (*ITO*) electrodes. This design achieved a detection limit of 211 fM with high target specificity [38]. Similarly, in situ growth methods allow the direct formation of QDs on conductive substrates, streamlining sensor fabrication. One notable example involved the in situ formation of *CdS/MoS_2_* heterojunctions on *ITO* via Cu^2+^-modulated nucleation, enabling PSA detection at 0.29 pg/mL in serum samples [64]. These scalable approaches support miniaturization and compatibility with portable and integrated diagnostic platforms.

### 7.5. One-Pot and Self-Assembly Strategies

One-pot synthesis offers an efficient, single-step route for generating functionalized QDs, minimizing the processing time and chemical waste. These methods are particularly useful in biomedical applications where streamlined fabrication is crucial.

A representative study demonstrated the one-pot synthesis of folate-functionalized carbon dots using *polydopamine* coatings. These nanodots enabled photothermal targeting of PSMA-positive PCa cells under near-infrared (NIR) irradiation [41]. Such simplified synthesis protocols reduce complexity while maintaining high functional specificity.

Self-assembly strategies further contribute to a modular sensor design. In one example, dual-color magnetic QD nanobeads were fabricated by layering green and red QDs onto magnetic cores, forming a lateral flow assay capable of simultaneously detecting free and complexed PSA [27]. These modular approaches facilitate multiplexing and allow rapid scaling for clinical and point-of-care deployment.

### 7.6. Surface Engineering and Layered Modification

Surface engineering plays a critical role in tuning the optical, electronic, and biochemical properties of QD-based systems. Techniques such as layer-by-layer deposition, ligand modification, and dopant incorporation can significantly improve biosensor sensitivity, selectivity, and biocompatibility.

**Figure 3:**
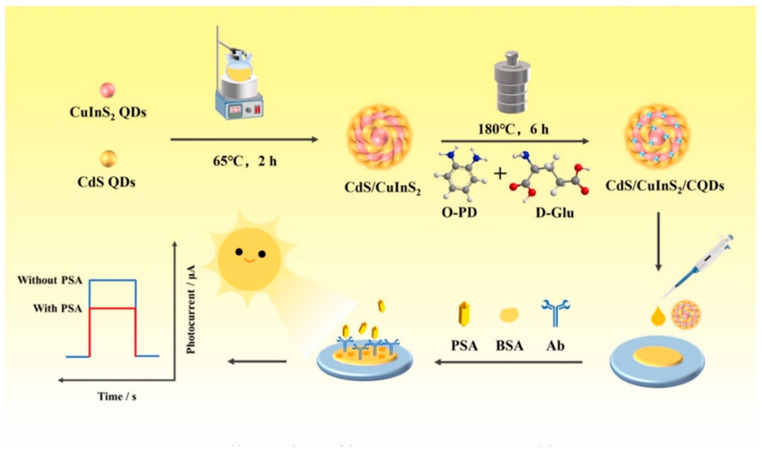
An Illustration of the immunosensor assembly process. Firstly, a *CdS/CuInS2/CQDs* suspension (3 mg/mL) was uniformly introduced guttatim to the electrodes and dried at 120 °C for 6 h. Afterward, 100 μL of PSA antibody (10 μg/mL) was fixed onto the surface of the *CdS/CuInS2/CQDs* electrodes. Next, 100 μL of 1 wt% bovine serum albumin (BSA) was added to the modified electrodes to eliminate the non-specific binding sites. Subsequently, 100 μL of PSA antigen was uniformly dropped onto the electrodes and incubated for 90 min to produce specific binding. To remove the excess free protein, the modified electrodes were rinsed with PBS buffer (0.01 mol/L) after each modification step. Adopted with permission from [48].

A notable example involves the *CQD* sensitization of combining semiconductor materials like cadmium sulfide and copper indium sulfide (*CdS/CuInS_2_*) heterojunctions. As illustrated in Figure 3, *CdS* and *CuInS_2_* QDs were synthesized and assembled into a heterostructure, which was subsequently modified with *CQDs* using a thermal treatment involving o-phenylenediamine (O-PD) and *D-glucose* (*D-Glu*). The resulting composite—*CdS/CuInS_2_/CQDs*—was drop-coated onto an electrode to fabricate a photoelectrochemical (PEC) immunosensor for PSA detection [48]. The sensor construction included the sequential immobilization of PSA-specific antibodies and bovine serum albumin (BSA) for nonspecific binding inhibition. Upon PSA binding, photocurrent changes under light irradiation were monitored. *CQD* sensitization enhanced both light harvesting and charge separation, resulting in up to a 10-fold signal amplification. This enabled ultrasensitive PSA detection in the femtomolar range in complex biological matrices. These layered, modular architectures are highly adaptable and hold significant potential for integration into multiplexed biosensing systems and multifunctional theranostic platforms.

The clinical utility of QD-based biosensors is intimately linked to the fabrication techniques employed. Methods such as hydrothermal synthesis [14,34,61], biogenic or green synthesis [34,54], and reverse microemulsion [2] provide reproducible and scalable production pathways with tunable optical and structural properties. Electrochemical deposition and in situ growth strategies [38] offer additional control over the sensor architecture and enable direct integration onto conductive platforms, supporting miniaturization and field deployment.

Complementary approaches such as one-pot synthesis [41], nanocarrier encapsulation [72], and layered heterojunction engineering [48,81] further expand the design space, introducing benefits such as simplified workflows, dual-mode therapeutic delivery, and improved signal amplification. Together, these fabrication strategies form a foundational toolkit for engineering QD-enabled biosensing systems that meet real-world diagnostic and theranostic demands.

## 8. Sensor Functionalization and Chemistry in PCa Biosensing

Sensor functionalization is a decisive factor in the overall performance of biosensors, especially in the context of PCa diagnostics where detecting low-abundance biomarkers with high specificity is essential. The integration of biorecognition elements, such as antibodies, aptamers, and oligonucleotide probes, onto QD-based or hybrid nanomaterial surfaces governs the sensitivity, selectivity, and signal transduction mechanisms of the biosensing platform. This section details the chemical functionalization strategies used in PCa biosensors for targeting both protein and nucleic acid biomarkers.

### 8.1. Antibody–Nanomaterial Conjugation for Immunosensing

The conjugation of antibodies to nanomaterials forms the basis of many QD-enabled immunosensors. Covalent binding methods, such as carbodiimide-mediated coupling using *1-ethyl-3-*(*3-dimethylaminopropyl*)*carbodiimide* (*EDC*) and *N-hydroxy succinimide* (*NHS*), or glutaraldehyde-based crosslinking, are widely applied to immobilize antibodies onto QD surfaces. These chemistries ensure the proper antibody orientation and preserve antigen-binding activity, which are essential for high-sensitivity and high-specificity detection.

A representative example of this strategy involves silica-coated *CdSe@ZnS* QDs functionalized with anti-PSA antibodies for use in lateral flow immunoassays (LFIAs). These particles yield bright and stable fluorescence signals, enabling efficient PSA detection in point-of-care settings [2].

**Figure 4:**
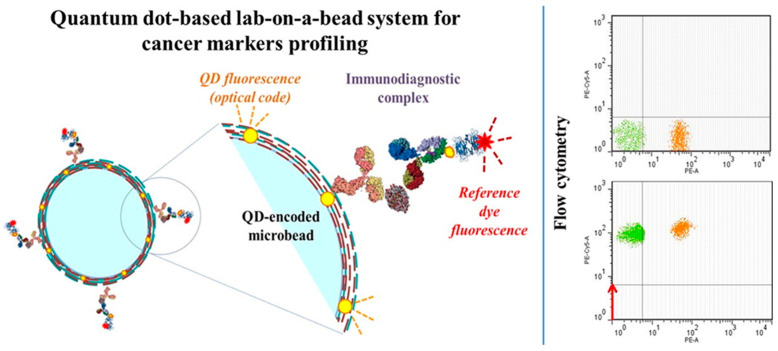
An illustration of the designed lab-on-a-bead system for the detection of prostate-specific antigen (PSA). Adopted with permission from [19].

For higher-throughput applications, QD-encoded microbeads have been integrated into suspension microarray platforms. As illustrated in Figure 4, this “lab-on-a-bead” design embeds multiple QDs with distinct emission wavelengths within a single microbead, facilitating the concurrent detection of multiple biomarkers such as total PSA and free PSA. Upon immunorecognition, *streptavidin*–Tri-COLOR conjugates bind to biotinylated detection antibodies, resulting in amplified fluorescence signals. Flow cytometry is then employed to differentiate the bead-associated fluorescence codes and quantify analyte levels [19]. This multiplexed immuno-sensing format improves the diagnostic throughput, supports biomarker profiling, and exemplifies the scalability of QD-based immunoassay technologies for clinical application.

Other approaches to antibody conjugation include vapor-phase glutaraldehyde crosslinking, which enables the stable attachment of antibodies on modified electrode surfaces. This method has been shown to enhance photocurrent generation in photoelectrochemical (PEC) biosensors, improving sensitivity for PCa biomarkers [75]. Additionally, sandwich-type immunosensors that combine magnetic bead-based target capture with QD fluorescence labeling have employed *hydrogen peroxide* (*H_2_O_2_*)*-*mediated signal amplification, achieving PSA detection at picogram levels [8]. These platforms illustrate the adaptability of antibody-based functionalization in both optical and electrochemical sensor formats.

### 8.2. Aptamer Functionalization and Surface Immobilization

Aptamers offer several advantages over traditional antibodies, including greater thermal and chemical stability, lower immunogenicity, and easier chemical modification. In QD-enabled biosensors, aptamers are often immobilized via thiol–gold (*Au–S*) chemistry, electrostatic adsorption, or π–π stacking interactions, allowing a precise orientation and preserving the structural integrity.

In one notable example, PSA-specific aptamers were immobilized on reduced graphene oxide functionalized with gold nanoparticles, while *Cu-doped carbon QDs* functioned as electron mediators. This hybrid electrochemical sensor achieved a detection limit of 3 pg/mL for PSA, combining sensitivity with label-free operation [10]. Another advanced platform integrated graphene QDs with *cobalt phthalocyanine* in a system that employed both differential pulse voltammetry (DPV) and electrochemical impedance spectroscopy (EIS), enabling PSA detection down to 0.66 pM [9]. These functionalization strategies support a modular design, multi-analyte capability, and robust signal transduction.

### 8.3. Nanocomposite Electrodes and Hybrid Architectures

To enhance the performance of QD-based biosensors, researchers have increasingly turned to nanocomposite electrodes that combine QDs with conductive polymers, metallic nanoparticles, and two-dimensional (2D) materials. These hybrid architectures enhance electron mobility, increase the electroactive surface area, and facilitate multiplexed or dual-mode sensing.

**Figure 5:**
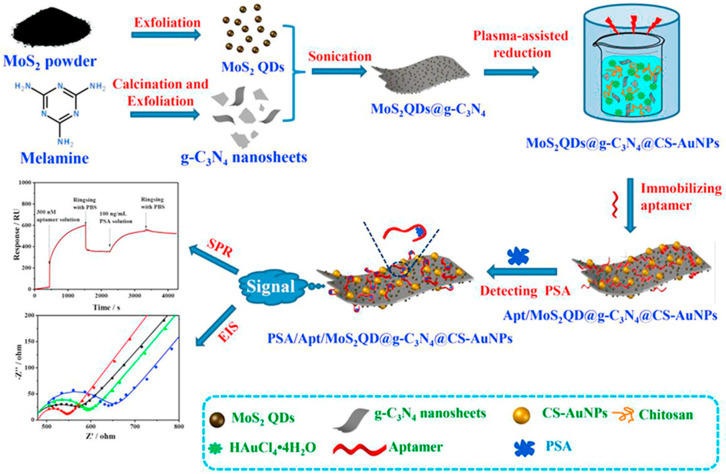
An illustration showing the principle of the constructed aptasensor based on the *MoS2QDs@g-C3N4@CS-AuNP* nanocomposite. Adopted with permission from [1].

A representative platform is illustrated in Figure 5, where a multifunctional aptasensor was constructed by co-assembling *MoS_2_* QDs, graphitic carbon nitride (g-C_3_N_4_) nanosheets, chitosan (CS), and gold nanoparticles (*AuNPs*) on a glassy carbon electrode [1]. The fabrication began with the exfoliation of *MoS_2_* powder to generate QDs and thermal decomposition of melamine to produce *g-C_3_N_4_* sheets. These components were then sonicated and combined, followed by plasma-assisted reduction with tetra chloroauric acid (*HAuCl_4_*) to anchor *AuNPs* onto the hybrid scaffold, forming the *MoS_2_QDs@g*-*C_3_N_4_@CS-AuNP* composite. The sensor was subsequently functionalized with a PSA-specific Aptamer, enabling selective binding. Dual-mode detection using electronic image stabilization (EIS) and surface plasmon resonance (SPR) demonstrated strong signal shifts upon target interaction, with a PSA detection limit as low as 0.71 pg/mL in serum [1]. As shown in the figure, both EIS and SPR provided distinct, quantifiable responses that confirmed the platform’s high analytical sensitivity.

In parallel, electro-chemiluminescence (ECL)-based systems have demonstrated complementary benefits. For instance, *carboxylate graphene QDs* (*CGQDs*) were employed in an ECL biosensor where the signal was quenched upon PSA binding, achieving detection limits of 0.29 pg/mL [47]. These architectures offer label-free, real-time biosensing options and are well-suited for clinical diagnostic applications.

Collectively, these advanced material combinations and surface chemistry strategies enhance the sensitivity, specificity, and adaptability of QD-based biosensors, paving the way for next-generation diagnostic tools in PCa care.

### 8.4. QD-Based FRET and ECL Biosensors

QD-based systems are inherently suited for Förster resonance energy transfer (FRET) and electrochemiluminescence (ECL) biosensing due to their size-dependent emission wavelengths, narrow spectral bandwidths, and exceptional photostability. These attributes facilitate high-sensitivity, multiplexed detection formats.

In a representative FRET immunoassay, QDs conjugated to anti-PSA antibodies functioned as energy acceptors from Lumi4-Tb donors in a time-gated detection setup. This system supported multiplexed PSA detection with emission peaks at 605, 655, and 705 nm, enabling the spectral discrimination of PSA isoforms. The platform achieved a detection limit of 2 nM [21], demonstrating the feasibility of QD-based FRET for clinical diagnostics.

ECL biosensors also benefit from QD integration, particularly when combined with signal amplification techniques such as rolling circle amplification (RCA). One ECL system employed aggregation-induced emission (AIE) polymer dots conjugated to antibodies, leveraging RCA to amplify the signal strength and spatial resolution. This platform enabled PSA detection down to 4.2 pg/mL while also providing imaging functionality [96].

These advanced systems rely on optimized bioconjugation strategies, including streptavidin–biotin coupling, DNA spacers, and linker optimization, to ensure efficient energy transfer, a precise molecular orientation, and high reaction kinetics. As a result, FRET and ECL biosensors based on QDs represent powerful tools for multiplexed and real-time analysis in PCa diagnostics.

### 8.5. DNA Hybridization Sensors for Nucleic Acid Biomarkers

Beyond protein detection, the identification of PCa-specific nucleic acid biomarkers, such as PCA3 and *TMPRSS2–ERG* gene fusions, has emerged as a key strategy for early diagnosis, risk stratification, and treatment planning. DNA hybridization-based biosensors are particularly effective at detecting these sequences with high specificity.

**Figure 6:**
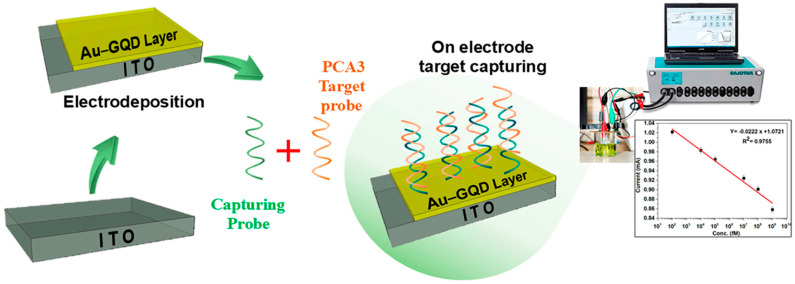
An illustration of the step-by-step fabrication of the proposed sensor for the PCA3 detection strategy showing indium–tin oxide (*ITO*) glass electrodeposited with *Au–GQDs*, followed by surface immobilization of ssDNA probes for capturing the target PCA3 and characterization by electrochemical studies (cyclic voltammetry (CV) and electrochemical impedance spectroscopy (EIS)). Adopted with permission from [38].

A compelling example is illustrated in Figure 6, where indium tin oxide (*ITO*) electrodes were modified via the electrochemical deposition of a gold–graphene QD (*Au–GQD*) nanocomposite [38]. This hybrid material provides a high surface area, excellent electrical conductivity, and biocompatibility, making it ideal for immobilizing single-stranded DNA (ssDNA) probes.

In this platform, ssDNA sequences complementary to the PCA3 transcript were covalently immobilized on the sensor surface. Upon hybridization with target nucleic acids, electrochemical responses were recorded via cyclic voltammetry (CV) and electrochemical impedance spectroscopy (EIS), yielding significant signal changes. The sensor achieved a detection limit of 211 fM with a total assay time of under five minutes.

This rapid, label-free approach offers a sensitive alternative or complement to PSA-based assays. By directly targeting molecular alterations specific to PCa, DNA hybridization biosensors represent a promising direction for personalized and early-stage diagnostics.

Sensor functionalization lies at the heart of nano biosensor performance in PCa diagnostics. Whether through covalent antibody attachment, thiol-mediated aptamer anchoring, or nucleic acid hybridization, these chemistries define biosensor sensitivity, specificity, and adaptability. QD integration further expands platform versatility, supporting multiplexed FRET and ECL detection, imaging capabilities, and point-of-care adaptability. As biosensing continues to evolve, these chemically engineered interfaces will play an increasingly central role in clinical translation and precision diagnostics diagnostics [1,2,8,9,10,19,21,38,47,75,96].

## 9. Quantitative Performance Metrics in PCa Biosensing

Quantitative performance metrics are essential in assessing the clinical viability of biosensors for PCa diagnostics. Metrics such as the limit of detection (LOD), linear dynamic range, specificity, reproducibility, and alignment with clinical standards like ELISA collectively determine the robustness and diagnostic relevance of a platform. QD-based biosensors have demonstrated substantial advancements across these criteria, particularly in applications involving total and free PSA detection.

### 9.1. Limit of Detection (LOD): Analytical Sensitivity

The LOD defines a biosensor’s ability to detect extremely low concentrations of a target biomarker, which is critical for early cancer detection. QD-enabled systems consistently achieve detection limits in the femtogram per milliliter (fg/mL) or even atto-gram per milliliter (ag/mL) range in complex matrices. For example, an electrochemiluminescence (ECL) immunosensor utilizing *GQD*-embedded *TiO_2_* nanotubes achieved a remarkable LOD of 1 fg/mL in serum samples [58]. A MoS_2_ QD-based impedimetric sensor detected PSA at 0.01 pg/mL with near-complete recovery in spiked serum [73]. Another dual-quenching ECL system that incorporated *graphitic carbon nitride QDs* and *Ag@MXene* hybrids reached an LOD of 6.9 fg/mL [66]. Additional examples include an Au/CQD-based aptasensor with 2 fg/mL sensitivity [30] and a *GQD–poly*(*thionine*) composite sensor that achieved 0.005 ng/mL detection in serum [11].

### 9.2. Linear Dynamic Range: Screening and Monitoring Capability

A broad linear dynamic range allows biosensors to function effectively across different diagnostic and treatment stages. QD-based systems offer substantial flexibility in this regard. One sensor demonstrated a linear range from 1 pg/mL to 100 ng/mL with a detection limit of 0.45 pg/mL [67]. Another hybrid platform extended the range from 1 to 3500 pg/mL, achieving a detection limit of 0.136 pg/mL [63]. Dual-range platforms have also emerged; for instance, a *GQD/poly*(*thionine*)*-*based sensor exhibited two dynamic zones from 0.0125 to 80.0 ng/mL, while maintaining a consistent limit of detection (LOD) of 0.005 ng/mL [11]. This adaptability across a wide concentration spectrum makes these sensors suitable for both early detection and longitudinal monitoring.

### 9.3. Specificity and Reproducibility in Complex Matrices

Specificity ensures that biosensors distinguish PSA from similar proteins and maintain signal accuracy in biologically complex environments. Reproducibility ensures consistent results across multiple samples or time points. QD-based systems have excelled in both. A smartphone-integrated lateral flow immunoassay (LFIA) using QD-embedded silica nanoparticles demonstrated no false negatives across 47 serum samples and retained stable signal output for over 10 days [4,69]. A turn-on fluorescence sensor using *GQDs@Ag* achieved an LOD of 0.3 pg/mL with excellent signal resolution in serum [8]. Aptamer-functionalized systems have shown similarly high specificity: a *GQD–CoPc* aptasensor reached a detection limit of 0.66 pM with negligible cross-reactivity [9], while a *nitrogen-doped GQD* platform detected PSA at 1.54 pM in spiked serum samples [68]. Furthermore, an impedimetric biosensor utilizing gold nanoparticles supported on or combined with a structure made of multi-walled carbon nanotube and graphene QD (*AuNP@MWCNT-GQD*) hybrids achieved 0.48 pg/mL sensitivity and matched ELISA performance across a wide 1–10,000 pg/mL dynamic range [59].

### 9.4. Validation Against ELISA and Clinical Standards

Clinical translation requires biosensors to match or exceed the performance of gold-standard assays such as ELISA and radioimmunoassay (RIA). Several QD-based systems have demonstrated excellent agreement with these reference methods. A QD-based microarray platform, designed as a “lab-on-a-bead”, simultaneously quantified free and total PSA levels in serum with ELISA-comparable accuracy while offering superior throughput [19]. A DNA barcode-enabled immunoassay using magnetic separation techniques achieved a 1 fg/mL LOD and demonstrated strong correlations with ELISA (r = 0.950) and RIA (r = 0.967) across 135 patient samples [97]. Dual-color magnetic QD nanobeads allowed the concurrent detection of free PSA at 0.009 ng/mL and complexed PSA at 0.087 ng/mL, aligning closely with established clinical benchmarks in the diagnostic gray zone [27].

### 9.5. Multiplexing and Imaging-Based Quantification

QD-enabled biosensors also offer unique advantages in multiplexed detection and imaging-based quantification, enhancing the diagnostic depth. One multianalyte platform simultaneously detected PSA (0.22 ng/mL), carcinoembryonic antigen (CEA, 0.56 ng/mL), and ATP (80 nM), demonstrating the cross-marker capability of QD-based designs [49]. For imaging applications, QD-labeled antibody probes targeting PSCA enabled intensity-based tumor grading with sustained fluorescence for up to four weeks [42]. Additionally, QD in situ hybridization (QD-ISH) assays targeting the *ERG* and *PTEN* genes were fully compatible with routine immunohistochemistry, supporting molecular-level diagnostics in tissue samples [20]. These innovations combine quantitative sensitivity with spatial resolution, contributing further to clinical utility.

## 10. Sample Type and Matrix Compatibility in PCa Biosensing

**Scheme 2:**
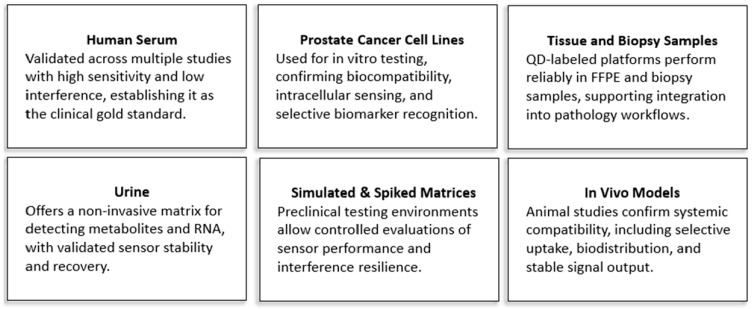
Summary of biological matrix compatibility in QD-based PCa biosensing platforms. Matrix types include human serum, PCa cell lines, formalin-fixed paraffin-embedded (FFPE) tissues and biopsies, urine, simulated/spiked media, and in vivo animal models. The figure aggregates reference sources supporting matrix-specific validation and performance metrics across diagnostic applications. References—Human Serum: [27,58,67,68,73,81,97,98]; PCa Cell Lines: [36,41,44,54,56,72,74,79]; Tissue & Biopsy Samples: [20,42,45,50,69]; Urine: [22,32,33,34]; Simulated & Spiked Matrices: [9,10,30,38]; In Vivo Models: [39,43,52].

The clinical utility of QD-based biosensors for PCa diagnostics is closely tied to their compatibility with a variety of biological matrices. These include serum, urine, tissue samples, cell cultures, and in vivo systems, each presenting specific challenges such as biomolecular complexity, variability, and matrix interference. Demonstrating biosensor performance across these diverse environments is essential for ensuring detection reliability, reproducibility, and eventual clinical translation. Scheme 2 illustrates the range of biological matrices, while Table 4 summarizes material designs and corresponding analytical performance.

### 10.1. Human Serum: The Clinical Standard

Human serum remains the gold standard matrix for diagnostic validation and is the most extensively studied medium for QD-based PSA detection. Multiple biosensors have demonstrated high recovery, a strong correlation with ELISA, and robust performance despite the presence of common interferents such as glucose, albumin, and uric acid. A sandwich-type electrochemical immunosensor utilizing QD-labeled secondary antibodies achieved a detection limit of 0.45 pg/mL and a linear range up to 100 ng/mL [67]. A *MoS_2_* QD-based platform demonstrated 0.01 pg/mL sensitivity and near-complete recovery of spiked PSA in serum [73]. DNA barcode-based immunoassays extended validation across 135 clinical serum samples, reaching 1 fg/mL sensitivity and aligning well with both ELISA and radioimmunoassay (RIA) results [97]. Additional validation has come from fluorescence sensors, aptamer-functionalized electrodes, and hybrid electrochemical platforms, all of which have shown strong specificity and reproducibility in serum [9,27,81,98]. Moreover, because serum is the most commonly accepted matrix in clinical diagnostics, biosensors validated in serum are better positioned for regulatory approval and clinical adoption.

### 10.2. PCa Cell Lines: Functional In Vitro Validation

PCa cell lines such as PC3 and lymph node carcinoma of the prostate (LNCaP) serve as critical models for evaluating biosensor biocompatibility, intracellular uptake, and therapeutic targeting potential. For instance, biogenically synthesized *CdTe* QDs selectively induced apoptosis in PC3 cells without affecting non-target cells [54]. QD probes directed against vascular endothelial growth factor receptor 2 (VEGFR2) achieved efficient uptake in LNCaP cells and increased tumor-associated fluorescence in both in vitro assays and xenograft models [74]. *GQDs* in conjunction with *CRISPR-Cas9* have been used to restore TP53 function in PC3 cells while sparing healthy cells from toxicity [79]. Additional applications include intracellular zinc ion (Zn^2+^) sensing and targeted drug delivery using QDs and carbon-based dots [36,41,56,72]. These findings support the dual roles of QD systems in diagnostics and therapeutics within cellular environments.

### 10.3. Tissue and Biopsy Samples: Histopathological Integration

QD-based biosensors are increasingly compatible with histopathological workflows, particularly through applications in immunohistochemistry (IHC) and in situ hybridization (ISH) on formalin-fixed, paraffin-embedded (FFPE) tissue samples. Immunolabeling of key biomarkers, such as prostate-specific membrane antigen (PSMA), prostate stem cell antigen (PSCA), and epithelial–mesenchymal transition (EMT)-related proteins, has demonstrated strong correlations with the tumor grade and stage [42,45,69]. QD-based ISH assays targeting *PTEN* and *ERG* gene fusions have shown stable signal retention across varying fixation times and diagnostic concordance with traditional IHC [20]. Furthermore, molecular quantification tools such as QD-based improved immuno quantitative analysis systems (QD-IIQAS) have enhanced the sensitivity of biomarker detection, such as for delta/notch-like epidermal growth factor-related receptor (DNER), offering quantifiable alternatives to conventional staining methods [50].

### 10.4. Urine: A Non-Invasive Diagnostic Matrix

Urine offers a convenient and non-invasive matrix for early PCa screening. QD-enabled biosensors have been applied to detect both metabolic and genetic biomarkers in urine. *Nitrogen-doped and triacylglycerol* (*TAG*)-functionalized carbon dots synthesized from natural materials successfully detected citrate in urine and live cell systems at concentrations as low as 3.5–4 nM [32,34]. A multicolor QD assay targeting *TMPRSS2–ERG* gene fusions identified these transcripts in urine and cell lysates with a detection limit of 1 fmol, performing comparably to RT-PCR-based methods [22]. These biosensors enable highly sensitive, accessible point-of-care diagnostics.

### 10.5. Simulated and Spiked Matrices: Preclinical Benchmarking

Simulated matrices, such as phosphate-buffered saline (PBS), redox buffers, and synthetic serum, are commonly used in early-stage testing to optimize biosensor design under controlled conditions. These matrices allow researchers to isolate and evaluate key performance characteristics before clinical validation. An *Au/CQD* hybrid sensor demonstrated a 2 fg/mL detection limit in *K_4_[Fe*(*CN*)*_6_]* buffer [30], while *Cu-doped carbon QDs* achieved 3 pg/mL sensitivity in redox-active media [10]. Other biosensors using *GQDs* and *cobalt phthalocyanine* achieved picomolar-level detection in both buffer and serum-spiked samples [9]. Electrodes modified with *Au–GQD* composites enabled PCA3 detection down to 211 fM [38]. These systems serve as essential bridges between the lab-based proof of concept and clinical application.

### 10.6. In Vivo Models: Systemic Validation

Although less commonly explored, in vivo studies are critical for assessing the biodistribution, targeting efficiency, clearance pathways, and imaging resolution. Folic acid–conjugated polymer QDs administered to severe combined immunedeficiency (SCID) mice selectively accumulated in lymph node carcinoma of the prostate (LNCaP) tumors within 60 min and were fully cleared via renal excretion within 3 h, demonstrating both effective targeting and minimal retention [43]. QD800 probes directed at PSMA facilitated the detection of micro metastases as small as 5000 cells, showing utility in intraoperative imaging [39]. Multiplexed QD labeling (MQDL) has enabled the single-cell imaging of signaling proteins such as c-Met and NF-κB in both xenograft models and human tissue samples, offering insights into dynamic biomarker expression for therapy monitoring [52].

QD-based biosensors have demonstrated high adaptability across a wide range of biologically relevant sample types. Serum remains the clinical benchmark, while urine offers a non-invasive screening alternative. Tissue samples support integration into standard histopathology, and PCa cell lines enable mechanistic explorations and therapeutic targeting studies. In vivo models provide crucial translational insights, and synthetic matrices serve as foundational tools for method optimization. This matrix versatility underscores the translational readiness of QD-enabled diagnostic systems for broad application in PCa detection and management. Table 4 provides a comprehensive summary of these matrix-specific innovations and analytical performances.

## 11. Benchmarking Nanomaterial-Enhanced Biosensors Against Traditional Assays

The body of comparative research clearly demonstrates that QD-based, and nanomaterial-enhanced biosensors offer significant performance advantages over conventional diagnostic techniques such as ELISA, surface plasmon resonance (SPR), and immunohistochemistry (IHC) for detecting PCa biomarkers. Numerous QD-based platforms have achieved detection limits in the sub-picogram to femtogram per milliliter range, far surpassing the typical ELISA threshold of approximately 1 ng/mL. For example, both the DNA barcode dot immunoassay [97] and the electrochemiluminescence (ECL) sensor based on *GQD/TiO_2_* nanotube arrays [58] reached detection limits as low as 1 fg/mL, representing over a million-fold sensitivity enhancement relative to traditional formats.

Electrochemical detection strategies, in particular, have consistently outperformed their optical counterparts. The *MoS_2_/g-C_3_N_4_* hybrid biosensor [1] demonstrated detection capabilities approximately 1000 times more sensitive than an SPR-based platform, while a label-free electrochemical immunosensor leveraging *GQDs* and *poly*(*thionine*) achieved a 200-fold improvement in sensitivity compared to fluorescence-linked immunoassays [11]. These dramatic gains underscore the unique transduction advantages provided by nanomaterial–electrode interfaces and signal-amplifying composites.

Importantly, the superiority of these biosensors is not confined to ideal laboratory conditions. Several platforms have been successfully validated in real clinical samples, supporting their translational viability. Studies, including [6,29,58,60,67], report high recovery rates, strong reproducibility, and robust concordance with established clinical assays. Notably, recent biosensors such as the *MXene–GQD* aptamer-based system [60] and the *Au/CQD* electrochemical sensor [30] have demonstrated performance metrics on par with or exceeding those of modern chemiluminescent immunoassays. These platforms not only offer increased sensitivity but also provide operational advantages such as a shorter assay time, simplified workflows, and enhanced portability.

Furthermore, innovations in signal conversion mechanisms have contributed to the expanding analytical capabilities of QD-enabled platforms. Approaches such as photoelectrochemical amplification [6,29,58] and nanohybrid-based signal quenching [60] have delivered improved signal-to-noise ratios and minimized background interference. These refinements are particularly beneficial in clinical contexts involving the early-stage diagnosis or patients presenting with low biomarker levels, where traditional assays may fail to detect the disease presence reliably. While achieving ultralow detection limits is a major technical advancement, it is important to note that sensitivity alone does not guarantee improved clinical outcomes. In certain contexts, detecting biomarkers at extremely low concentrations may contribute to overdiagnosis or unnecessary intervention. Therefore, for practical clinical utility, biosensors must also demonstrate high stability, reproducibility, and operational simplicity—factors equally critical to their successful adoption in routine diagnostics.

A comparative summary of these findings is compiled in Table 5, which outlines the detection limits, performance improvements over conventional platforms, and the degree of clinical validation. Together, this data provides compelling evidence that QD-based and nanomaterial-enhanced biosensors are not only scientifically robust but also clinically viable. Their adoption has the potential to transform PCa diagnostics by offering earlier detection, greater assay flexibility, and improved diagnostic precision.

## 12. Integrated Diagnostic Ecosystem for QD-Based PCa Detection

The clinical translation of QD-based biosensors depends not only on analytical sensitivity and specificity but also on their integration into a broader diagnostic ecosystem. This ecosystem includes advanced imaging technologies, signal processing methods, portable interface platforms, and software-assisted optimization tools. Together, these components form the foundation for scalable, reproducible, and context-appropriate diagnostic solutions, extending from centralized hospital laboratories to remote point-of-care (POC) environments.

### 12.1. Imaging Systems for Multiplexed Biomarker Detection in Prostate Tissue

High-resolution and multiplexed imaging platforms are essential for advancing PCa diagnostics, particularly for tissue-based biomarker analysis and prognostic stratification. QD-based fluorescence imaging systems, such as the Nuance multispectral imaging system, enable the spectral deconvolution of overlapping QD emission signals. This capability allows for the simultaneous visualization of multiple biomarkers within a single tissue section. For example, this approach has been used to quantify Akt-1 expression—a biomarker associated with disease recurrence and treatment resistance—in PCa tissue [26]. The integration of QD-based imaging into automated histopathology platforms like the Benchmark ULTRA has further enabled high-throughput immunohistochemistry workflows that align with clinical and regulatory standards, supporting widespread scalability and clinical adoption [15].

**Figure 7:**
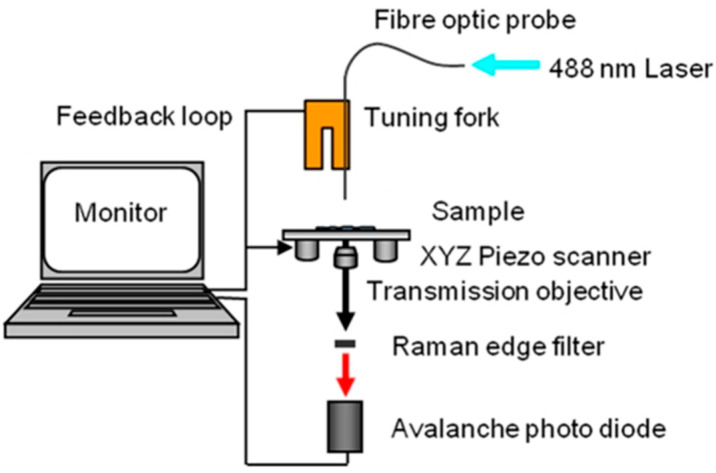
A Schematic of the SNOM experimental arrangement. Adopted with permission from [46].

Beyond traditional fluorescence imaging, near-field optical techniques provide even greater spatial resolution for nanoscale biomarker mapping. As shown in Figure 7, scanning near-field optical microscopy (SNOM) uses a 488 nm laser to excite a fiber optic probe, which is mounted on a tuning fork for precision control. The probe scans the QD-labeled sample on a piezoelectric XYZ stage while maintaining close proximity through a feedback loop. Emission signals pass through a Raman edge filter and are detected by an avalanche photodiode, allowing the ultra-resolution mapping of QD-tagged biomolecules [46].

This SNOM–QD integration has proven valuable in identifying subcellular protein distribution patterns, such as E-cadherin localization, which differ between localized and metastatic PCa phenotypes. By resolving protein distributions at the nanoscale, SNOM-assisted imaging has revealed distinct intracellular biomarker signatures that correlate with disease progression and metastatic potential [46]. These advanced imaging modalities expand the diagnostic value of QDs beyond simple detection, offering spatial and mechanistic insights critical for precision oncology.

### 12.2. Analytical Platforms for Quantifying QD Behavior and Biomarker Interactions

Comprehensive physicochemical characterization is critical to ensure that QD-based biosensors meet the stringent standards required for clinical application, including stability, reproducibility, and biocompatibility. Techniques such as UV–Vis spectroscopy, fluorescence lifetime imaging, and photoluminescence profiling are widely used to confirm the emission characteristics and evaluate surface quality [32,34,53]. Additional tools like Fourier-transform infrared spectroscopy (FTIR), X-ray diffraction (XRD), and transmission electron microscopy (TEM) provide structural and morphological insights that guide sensor development.

These analytical methods have direct implications for biosensor performance. For instance, zinc selenide (selenium) sulfide [ZnSe(S)] QDs synthesized via microwave-assisted techniques were shown to possess well-controlled surface defects and minimal cytotoxicity in PC-3 PCa cells, supporting their use in theranostic platforms [53]. Flow cytometry has also been adapted to enable the high-throughput quantification of free and complexed PSA using QD-labeled microbeads. Compared to traditional ELISA, this approach offers enhanced sensitivity and a reduced assay time, making it suitable for large-scale clinical screening [19,23].

### 12.3. Electrochemical Signal Processing for Quantitative PSA Detection

Electrochemical signal transduction remains a cornerstone of QD biosensing due to its high sensitivity, low sample requirements, and compatibility with miniaturized diagnostic systems. Methods such as cyclic voltammetry (CV), square wave voltammetry (SWV), and electrochemical impedance spectroscopy (EIS) are commonly used to quantify PSA levels and monitor aptamer–target interactions on sensor surfaces [1,9,10,29,38].

A notable example is a biosensor utilizing a graphene–gold electrode modified with *TiO_2_/carbon QDs*, which achieved a detection limit of 0.007 ng/mL under optimized electrochemical conditions, enabling differentiation between free and total PSA in complex matrices [3]. In another study, photoelectrochemical (PEC) platforms employing *CdS/MoS_2_*-coated indium tin oxide (*ITO*) electrodes achieved PSA detection at 0.29 pg/mL [64], while electro-chemiluminescent (ECL) sensors based on *GQD/TiO_2_* composites demonstrated ultralow detection thresholds of 1 fg/mL in serum [58]. These approaches demonstrate the feasibility of high-performance PSA detection within compact, reproducible formats suitable for clinical use.

### 12.4. Smartphone-Compatible and Portable Diagnostic Interfaces

A major advancement in QD applications is their integration into smartphone-compatible and portable diagnostic systems designed to meet the growing need for decentralized, point-of-care testing. These platforms combine the unique optical and electronic properties of QDs with mobile technologies to provide real-time, low-cost cancer detection and monitoring, which are particularly valuable in resource-limited or remote settings.

A wide spectrum of QD-enhanced biosensing formats has been developed, including lateral flow immunoassays [4,27], paper-based and colorimetric assays [7], electrochemical and fluorescent immunosensors [11,101,102], and ratio-metric fluorescence nanoprobes [103,104]. These systems often leverage smartphone cameras, wireless signal modules, or app-based image processing to quantitatively interpret diagnostic signals, enabling lab-grade analysis in field environments.

To further increase portability and accessibility, QD-based biosensors are now being designed with integrated modules such as magnetic preconcentration units, electrochemical detectors, and mobile app connectivity. For example, a dual-mode paper-based immunoprobe using sulfur-doped carbon dots achieved PSA detection limits of 38 pg/mL in electrochemical immunoassay (ECIA) mode and 5 ng/mL in fluorescence immunoassay (FIA) mode, with high reproducibility in serum samples [27,61]. Another study reported a lateral flow assay incorporating QD-silica nanoparticles that yielded zero false negatives across 47 clinical serum samples and achieved a detection limit of 0.138 ng/mL, maintaining strong fluorescence stability for a post-assay analysis [4].

These innovations are not only accurate but also user-friendly, making them ideal for home diagnostics, telemedicine, and remote healthcare delivery. QD-based diagnostic systems have successfully targeted a broad range of cancer biomarkers, including PSA [4,7,27,61,102], carcinoembryonic antigen (CEA) [7], BRCA-1 gene sequences [105], Fe^3+^ ions in tumor environments [106], and even whole cancer cells [101]. Many of these systems achieve ultrasensitive detection limits in the nanogram per milliliter or picomolar range.

Smartphone integration facilitates signal quantification through an RGB image analysis or software algorithms, removing the need for bulky or complex laboratory instruments. This capability supports real-time decision-making and rapid diagnostics directly at the point of care [4,7,103,105,106,107,108].

In advanced formats, QD-based sensors are embedded in responsive hydrogel matrices for dynamic monitoring of the tumor microenvironment. For example, pH-sensitive or strain-responsive hydrogels loaded with carbon dots can detect acidity or pressure fluctuations and wirelessly transmit this data to smartphones [104,107]. This enables continuous, non-invasive monitoring without the need for clinical infrastructure.

Additionally, some biosensors use smartphone-based platforms for detecting cancer-associated enzymes [101], further broadening their diagnostic reach. These mobile-compatible systems are particularly significant in global health contexts, offering cost-effective, portable, and reliable solutions for disease detection and longitudinal patient monitoring.

### 12.5. Software Tools for Assay Optimization and Image Processing

The deployment of QD-enabled diagnostic systems is significantly enhanced by software tools that facilitate assay optimization, improve reproducibility, and support automated image analysis. Statistical software platforms such as Design Expert employ design-of-experiment (DoE) methodologies to systematically optimize variables such as reagent concentrations, incubation times, and transduction parameters, ultimately boosting assay robustness and sensitivity [3].

In imaging applications, custom algorithms and digital processing tools have been developed to support QD-based diagnostics. Techniques such as top-hat transformation and dot-pattern energy mapping have improved edge detection and signal clarity in hybrid imaging systems that combine QD fluorescence with transrectal ultrasound (TRUS), particularly in complex tissue environments [25]. These software routines not only reduce inter-operator variability but also enable the consistent and scalable interpretation of imaging data.

In sum, the analytical and computational systems supporting QD-based biosensors form a synergistic diagnostic ecosystem. High-resolution imaging benefits from algorithm-driven analysis; point-of-care devices leverage robust electrochemical detection and smartphone interfaces; and biosensor development is guided by simulation-informed assay optimization. This comprehensive integration across material science, signal transduction, hardware engineering, and software analytics is essential for transitioning QD-based biosensors from experimental tools to clinically regulated, scalable diagnostics for PCa.

## 13. Limitations and Future Directions for QD-Based PCa Diagnostics

Despite promising advancements, QD-based biosensors for PCa diagnostics face significant challenges that limit their clinical translation. These challenges span clinical, technical, and regulatory domains, creating a gap between experimental success and real-world deployment.

A primary limitation is the lack of robust clinical validation. While many QD-enabled platforms demonstrate high sensitivity—often at the femtogram level—in buffer systems or spiked samples, few have been rigorously tested using clinical specimens or integrated into standardized diagnostic workflows [4,5,10,11,14,19]. Some early-stage studies show encouraging results in serum or tissue samples, yet most technologies remain at the proof-of-concept stage, without progression to clinical trials or regulatory review.

Another concern is the limited evaluation of long-term operational stability and sensor reusability. Although some biosensors retain function for up to 10 days [4], few studies assess performance across multiple operational cycles, storage temperatures, or clinical handling protocols—key factors for real-world deployment [3,10,67,73]. This shortfall is especially problematic for point-of-care (POC) or decentralized diagnostics, where robustness and reliability are critical.

Validation across complex biological matrices remains incomplete. While serum is the most commonly tested fluid, relatively few systems have been evaluated in undiluted urine, whole blood, or tissue homogenates. These matrices pose unique challenges, including protein fouling, pH variability, and autofluorescence, that can impair analytical performance [7,22,38]. Systematic studies are needed to confirm matrix compatibility and analytical robustness in clinically relevant environments.

The diagnostic scope of current QD-based sensors is also limited. Most focus on traditional markers like PSA, with few platforms demonstrating the true multiplexed detection or quantitative analysis of emerging biomarkers such as PCA3, PSCA, or *TMPRSS2–ERG* [47]. Expanding biomarker panels could significantly improve the diagnostic specificity, enable disease staging, and support personalized treatment decisions [1,6,63].

Scalability presents another barrier. The multistep synthesis and modification of QDs, including doping, surface encapsulation, and bioconjugation, are often labor-intensive and not optimized for high-throughput or low-cost production [5,27,66,75]. Simplifying fabrication, adopting scalable formats like screen-printed electrodes or paper-based substrates, and integrating smartphone-readout technologies could enhance manufacturability and facilitate adoption in low-resource settings [8,21,33,65,77].

Biosafety and environmental toxicity further complicate clinical translation. Many QD systems rely on hazardous elements like cadmium, mercury, or selenium [16,17,39,42,69,96]. While interest is growing in biocompatible alternatives, such as carbon, silicon, or indium-based QDs, few platforms fully embrace these safer materials [8,10,23,73,81]. Long-term in vivo safety and the environmental fate remain underexplored, hindering regulatory approval and ethical deployment.

Batch-to-batch variability also requires attention. Most studies report results from a single batch of QDs, without addressing synthesis reproducibility or performance consistency across multiple lots [30,67,75]. This lack of quality control is a major obstacle to clinical scale-up and standardization.

Imaging-based applications, while strong in tumor localization, often lack quantitative outputs. Essential metrics like calibration curves, detection limits, or correlations with clinical benchmarks are frequently missing, limiting their utility in comparative diagnostics [7,22,38].

To overcome these hurdles, future efforts should align technical innovations with practical, regulatory, and clinical needs. Priorities include expanding biomarker targets for multiplexed detection, validating performance across complex biological samples, improving sensor stability and reusability, and simplifying fabrication for scalable, low-cost production. Importantly, transitioning to cadmium-free and environmentally responsible materials will be crucial for regulatory success and patient safety.

QD-based biosensors that achieve multiplexing, matrix robustness, scalable manufacturing, and biocompatibility will be best poised for clinical translation. By integrating these advances, next-generation platforms can fulfill the unmet needs in the early detection, monitoring, and personalized management of PCa.

## 14. Conclusions

QD-based biosensors have emerged as powerful diagnostic tools for prostate cancer, offering ultralow detection limits, broad dynamic ranges, and versatile signal transduction modalities. This review has illustrated how QDs, when integrated with carbon-based nanomaterials, metal nanoparticles, and hybrid composites, deliver exceptional sensitivity across electrochemical, fluorescence, and photoelectrochemical platforms.

These systems are uniquely positioned to address critical limitations in conventional diagnostics, including the low specificity of PSA testing and the reliance on invasive procedures. Through multiplexed detection of PSA isoforms, gene fusions (such as *TMPRSS2–ERG*), and metabolic biomarkers, QD-enabled biosensors support more accurate disease detection, staging, and therapeutic monitoring. Their demonstrated compatibility with a range of biological matrices, including serum, urine, tissue samples, and in vivo models, further highlights their translational potential.

To realize their full clinical impact, the next generation of QD-based diagnostics must prioritize robust validation in real-world conditions, long-term sensor stability, scalable and cost-effective fabrication, and the adoption of cadmium-free, biocompatible materials. Coupling these systems with portable readout devices and AI-driven signal analysis will also be key to enabling decentralized, point-of-care deployment.

As these technologies move from laboratory innovation to clinical integration, QD-based biosensors are poised to transform prostate cancer diagnostics, enabling earlier detection, reducing overtreatment, and facilitating personalized care with an unprecedented level of precision.

## Figures and Tables

**Table 2:** Functional roles and signaling mechanisms of QD-based nanoplatforms in biosensing and imaging.

Nanostructure Modality/Platform Design	Detection Principle and Nanomaterial Functionalization	Biomedical Utility and Application Scenario	Supporting Studies
Quantum Dots as Core Materials	QDs (e.g., *CdSe*, *CdTe*, *GQDs*, *MoS_2_*) are used as fluorophores or electron mediators and functionalized for biorecognition	Foundational components for both optical and electrochemical biosensors	[1,3,21,40,74]
Fluorescence-Based Detection	FRET, confocal, and two-photon techniques are enabled by surface-modified QDs	Enables sensitive biomarker detection, live-cell imaging, and tumor visualization	[4,21,40,44,52,74]
Electrochemical and Photoelectrochemical Detection	Cyclic voltammetry (CV), differential pulse voltammetry (DPV), electrochemical impedance spectroscopy (EIS), and photoelectrochemical (PEC) signals are enhanced by QD composites (e.g., *GQDs-AuNPs*); immobilize bioreceptors.	Provide ultrasensitive PSA detection in serum and lab-on-chip assays	[3,9,10,11,64,75]
Antibody/Aptamer Biointerfaces	Covalent or affinity functionalization of QDs with antibodies/aptamers targeting PSA, PSMA, and VEGFR2	Ensure target-specific recognition in biosensing and targeted delivery	[3,6,30,40,60,74]
Hybrid QD Composites	QDs are combined with *AuNPs*, *graphene*, *TiO_2_*, or *g-C_3_N_4_* to enhance charge transfer and stability	Improve the sensitivity and performance of multifunctional biosensors	[1,3,29,47,76,77]
Multiplexed/Multicolor Detection	QDs with discrete emissions are used to identify multiple targets in a single assay (e.g., PSA + CEA)	Enables parallel biomarker profiling for comprehensive diagnostics	[7,20,21,22,27,45]
Fluorescence Quenching/Recovery	Target-induced changes in QD emission via FRET or electron transfer mechanisms	Supports the reversible and real-time monitoring of biomolecular interactions	[16,33,49,60,65,78]
In Vivo Imaging and Theranostics	Functionalized QDs (e.g., anti-PSMA and FA) for tumor targeting and visualization in living systems	Facilitates non-invasive imaging and surgical guidance in cancer models	[39,40,41,43,46,74]
*Carbon-Based* and *g-C_3_N_4_ QDs*	Eco-friendly QDs synthesized from *carbon* or *g-C_3_N_4_*, offering photostability and tunability	Applied in green diagnostics, cellular imaging, and portable biosensors	[1,35,48,55,77,79]
Gene-Level Biosensing Platforms	QDs conjugated with DNA/RNA probes for ISH, hybridization assays, and mutation detection	Enables gene-level diagnostics of fusion events (e.g., *TMPRSS2-ERG*) and precision medicine	[15,20,22,38]

**Table 4:** Clinical matrix compatibility of QD-based biosensors: applications across serum, urine, tissues, and in vivo systems.

Biological Matrix/Sample Type	Detection Chemistry and Analytical Validation	Clinical Relevance and Translational Commentary	Representative References
Serum-Compatible Platforms	QD-based sensors for PSA and other biomarkers validated in human serum, including undiluted and spiked samples. Functionalization includes antibodies (e.g., sandwich assays and lateral flow immunoassays (LFIAs)), aptamers, and electrochemical probes. LODs were achieved from fg/mL to low ng/mL.	Serum validation indicates high translational value for clinical diagnostics. The high sensitivity in complex biological fluids demonstrates robustness for routine lab or POC use.	[3,4,8,11,19,58,59,60,75,98]
Urine-Compatible and Non-Invasive Platforms	Applied to citrate and gene detection in human urine using QD, CD-based fluorescence quenching, and hybridization probes. Assays show high recovery and selectivity.	Urine-based sensors enable non-invasive cancer monitoring. Matrix tolerance broadens applicability for home testing or early screening.	[22,32,33,34]
Cell Lysates and Live Cell Systems	Sensors and probes validated in PCa cell lysates (PC3 and LNCaP) for PSA, PCA3, Zn^2+^, or the therapeutic response. QDs are used in aptasensor, *CRISPR* delivery, and intracellular imaging.	Compatibility with cell-derived matrices supports applications in research, drug screening, and personalized therapy development.	[6,17,36,37,79,99]
Tissue-Based and FFPE-Compatible Platforms	QDs are used for immunolabeling PSCA, EMT markers, *PTEN/ERG* genes in FFPE tissues or biopsies. The assays outperform IHC in fluorescence durability and multiplex imaging.	Enable high-resolution, retrospective diagnostic imaging. Support biomarker quantification in pathological workflows.	[15,20,26,42,45,69,100]
In Vivo and Animal Model Validated Platforms	In vivo imaging of prostate tumors (xenografts and tibial implants) using QDs conjugated to PSMA, folate, and other ligands. Biodistribution, fluorescence tracking, and targeting specificity are confirmed.	Demonstrate potential for real-time diagnostics and image-guided therapy in oncology. Support preclinical evaluations of targeting and delivery strategies.	[39,40,43,57,74]
Matrix Interference-Resistant Systems	Systems were explicitly assessed with glucose, BSA, L-cysteine, etc., in serum or buffer matrices. Maintained high selectivity and stability. LODs achieved in the low pg/mL to pM range.	These platforms offer resilience in complex clinical samples, reducing false positives and improving reliability in uncontrolled environments.	[9,30,33,49,68,73,81]

**Table 5:** Comparative performance of new vs. traditional methods.

New Method	Target Analyte	Detection Limit (LOD)	Performance Improvement	Comparison Basis	Reference
Electrochemical aptasensor	PSA	0.028 ng/mL	~35× more sensitive than ELISA	Compared to ELISA (~1 ng/mL)	[3]
Photoelectrochemical aptasensor	PSA	0.52 pg/mL	~2000× improvement	Direct comparison to ELISA in serum	[6]
Label-free electrochemical sensor	PSA	0.005 ng/mL	~200× improvement	Validated against an enzyme-linked fluorescence assay	[11]
Bifunctional biosensor (*MoS_2_/g-C_3_N_4_*)	PSA	0.71 pg/mL	~1000× lower LOD than SPR (0.77 ng/mL)	Head-to-head comparison with SPR	[1]
Enzyme-free sandwich electrochemical sensor	PSA	0.45 pg/mL	High sensitivity	Validated against reference clinical methods	[67]
DNA barcode dot immunoassay	PSA	1 fg/mL	>1,000,000× improvement	Correlated with ELISA and RIA	[97]
Electrochemical *Au/CQD* aptasensor	PSA	2 fg/mL	Ultrasensitive vs. conventional methods	Compared to ELISA (typical LOD ~1 ng/mL)	[30]
*GQD/TiO_2_* nanotube ECL immunoassay	PSA	1 fg/mL	Extremely high sensitivity	Strong agreement with chemiluminescent methods	[58]
*AuNS/S-GQD* electrochemical immunosensor	PSA	9.7 aM (buffer), 26 fg/mL (serum)	One of the lowest reported LODs	Validated in serum with 97–107% recovery	[29]
*MXene-GQD* fluorescence aptasensor	PSA	0.03 ng/mL	Similar to chemiluminescent immunoassays	Validated in human serum samples	[60]
